# Classifying marine mammals signal using cubic splines interpolation combining with triple loss variational auto-encoder

**DOI:** 10.1038/s41598-023-47320-4

**Published:** 2023-11-15

**Authors:** Nhat Hoang Bach, Le Ha Vu, Van Duc Nguyen, Duy Phong Pham

**Affiliations:** 1 Institutes of Science and Technology, Institute of Electronics, Hanoi, 10000 Vietnam; 2 School of Electricity-Electronics, Hanoi University of Science and Technology, Hanoi, 10000 Vietnam; 3 Faculty of Electronics-Telecommunications, Electric Power University, Hanoi, 10000 Vietnam

**Keywords:** Electrical and electronic engineering, Marine biology, Physical oceanography

## Abstract

In practical applications of passive sonar principles for extracting characteristic frequencies of acoustic signals, scientists typically employ traditional time-frequency domain transformation methods such as Mel-frequency, Short time Fourier transform (STFT), and Wavelet transform (WT). However, these solutions still face limitations in resolution and information loss when transforming data collected over extended periods. In this paper, we present a study using a two-stage approach that combines pre-processing by Cubic-splines interpolation (CSI) with a probability distribution in the hidden space with Siamese triple loss network model for classifying marine mammal (MM) communication signals. The Cubic-splines interpolation technique is tested with the STFT transformation to generate STFT-CSI spectrograms, which enforce stronger relationships between characteristic frequencies, enhancing the connectivity of spectrograms and highlighting frequency-based features. Additionally, stacking spectrograms generated by three consecutive methods, Mel, STFT-CSI, and Wavelet, into a feature spectrogram optimizes the advantages of each method across different frequency bands, resulting in a more effective classification process. The proposed solution using an Siamese Neural Network-Variational Auto Encoder (SNN-VAE) model also overcomes the drawbacks of the Auto-Encoder (AE) structure, including loss of discontinuity and loss of completeness during decoding. The classification accuracy of marine mammal signals using the SNN-VAE model increases by 11% and 20% compared to using the AE model (2013), and by 6% compared to using the Resnet model (2022) on the same actual dataset NOAA from the National Oceanic and Atmospheric Administration - United State of America.

## Introduction

At the UN’s Fifteen Informal Consultation on Oceans and Law of the Sea, held in New York, the Member States affirmed the role of marine life in the development of the world in general and of fisheries in the global food security policy in particular, and at the same time raised awareness of the risks that threaten marine life. On February, 2019, the Directorate of Fisheries of Vietnam cooperated with the Center for Marine Life Conservation and Community Development to organize a workshop on “Training on the identification of shark mammals named in the Cites Appendix for management, inspection and control”, which aimed to focus on solutions to manage and control shark mammals in protection list. On December, 2021, the Ministry of Natural Resources and Environment of Vietnam issued Document No.4944 reporting the status of the National Sea and Island Environment for the period 2016–2020, focusing on the assessment of marine environment and the diversity of marine life, as well as difficulties and advantages in marine research. From there, we see the urgency of building a management model capable of automatically classifying sound sources from marine creatures, not only in Vietnam but also in other countries, to manage and protect red-listed marine mammals from the effects of environmental change and human impact.

As marine mammals can hear a wide range of sound waves from infrasound to ultrasound, it is unavoidable that both civilian and military maritime activities can have negative effects on marine life. Controlled exposure studies involving the use of the US Navy’s SURTASS sonar system to investigate the behavioral responses of low-frequency hearing cetaceans have attracted scientists’ interests^1,2^. The results show that inadvertently generating acoustic signals with the same frequency range as cetacean hearing, even though the structures of those signals are different, can affect the responses of marine mammals to each other and to the surroundings. Some hydrological studies of marine environment and marine experiments in the bays of Vietnam have also recorded cases where marine animals followed ships and observation equipment that used sonar principles. Therefore, the timely detection and classification of marine mammal signals are extremely important to oceanography in general and underwater signal processing in particular.

Usually the algorithms used to detect and classify signals generated by marine mammals are based on methods of analyzing signal properties, and are generally divided into two main types of approaches:

- Approaches that involve comparing unlabeled data with labeled data. The commonly used method is matched filter, in which a sample corresponding to the signal to be processed is transformed with a reference signal combined with a threshold to conclude the desired signal^3,4^. The results of this method have been evaluated and tested on a Ultra short baseline (USBL) system that the research team has deployed to detect and manage moving underwater objects in practice. Another solution is spectral correlation, which first multiplies the correlation using segments of the spectral images, and then compares the result with a spectrogram of the unlabeled data, to generate a feature vector for similarity over time. The magnitude of the result corresponds to the detection ability.
- Approaches by detecting the desired region in a spectrogram and extracting features (such as detection time or feature frequencies) to use as the vector for classification purposes. One of the effective processing methods is to use mixed Gaussian model and hidden Markov model for classification^5,6^. Commonly used detection algorithms include: neighborhood searching in filtered, smoothed, and transformed spectrograms^7^ and contour detection for determining peaks in selected frequency bands of a normalized signal spectrum^8^. The accuracy of this solution depends on filtering, normalizing and smoothing the spectrogram. Once the desired region is determined, the feature vectors are fed into classifiers such as: linear discriminant analysis^9^, support vector machines^10,11^ and artificial neural networks^12–14^ and^15^. Our reseach focuses on this type of approaches.

Current research on directly using artificial intelligence (AI) to classify underwater acoustic signals as marine mammal signals often overlook the complex temporal variations of the acoustic channel. This will affect the quality of assessments in some classification tasks related to the statistical variability of the marine environment. If these important variables are ignored, the classification system will expose limitations in cases that require high reliability, such as in defense underwater structure systems, managing the fisheries, etc. When directly using AI methods and transfer learning to identify all the signal’s features from the sound source, the identification also process requires complex hardware structures and large computational loads to process the data. Therefore, adding a pre-processing step helps the classification model extract fundamental features from the overall data spectrum, thereby being able to highlight important features and reduce the computational complexity of the classification system.

In the past 10 years, methods combining pre-processing and artificial intelligence have achieved positive results in classifying marine organisms’ communication signals. Some results using machine learning can be listed as: classification of humpack whales using a trio of spectrogram image processing, Principal Component Analysis (PCA), and statistical cluster analysis classification of 34 species of beluga using Classification and Regression Tree (CART) and random forest or time-frequency domain processing based on matched filter^16^, spectrogram correlation^17–19^, MFCC-Gabor filter^20^. Recently, advancements in deep learning algorithms have opened up new approaches in underwater signal processing. After pre-processing signals in the form of spectrograms as input to Convolution Neural Networks (CNN), model training or learning transfer with pre-trained model weights is used to classify marine mammals. Another solution is to use Recurrent Neural Network (RNN) to extract features based on the instantaneous information obtained from the raw signal^21,22^.

Even though CNN and RNN models have shown remarkable results in many classification tasks, there are still limitations due to the requirement of a relatively large amount of data. Recently, a solution using Siamese Neural Network (SNN), with the purpose of classifying audio recordings according to the passive sonar principle, was introduced^23^ to reduce the model complexity but still guarantee the quality of classification results. A model using SNN deep learning model with data feature extraction^24^ was applied to explain the meaning of audio sequences. Besides, SNN was also used to classify sound sources^25,26^ instead of CNN thanks to its advantage in generalizability. Model^27^ used some clustering approaches to produce results on dissimilarity spaces, then used SVM for classification. Model^28–30^, transformed spectral images into a set of feature vectors for each sample to feed into SVM. The results show that this approach works better and is more flexible than typical CNN. Underwater signals can come from artificial, biological, as well as environmental sources, etc., and thus vary in length, intensity and frequency distribution. Bioacoustic signals are usually below 15 kHz, artificial signals from ships are mainly below 1 kHz, and environmental signals are often in the range of 100 Hz to 50 kHz^31^. In practice, a data set containing all these three types of signals can be obtained, that is, there can certainly be frequency overlap between the sound sources. Environmental signals typically have no dominant frequency, while artificial signals, which are the combination of wide-band and narrow-band signals, often have specific frequencies that are easier to detect compared to other frequencies. Such environmental factors and complexity will make the classification of underwater sound sources much more difficult.

Therefore, it is necessary to introduce a classification model with a non-complicated network structure that is capable of detecting and classifying different types of biotic and abiotic underwater signals on complex background noise. In this study, the proposed model uses STFT-CSI, MFCC and Wavelet to transform raw audio data into red (R), green (G) and blue (B) spectral images, respectively, and then stacks each set of three images into one RGB-image before feeding into an Siamese Neural Network-Variational Auto Encoder (SNN-VAE) network to classify marine mammals sound sources. The solution of using data interpolation does not increase the amount of useful information but rather focuses on highlighting the important features of the data. In this case, the interpolation algorithm functions similarly to a digital signal filter for extracting salient features of the dataset before subsequent steps of a classification system. Besides that, the hidden space of VAE is constrained to be smooth and continuous, limiting the impact on the reconstruction of the output. The results of the proposed solution show that the model is capable of classifying communication signals of marine mammals with 90% accuracy even with small datasets and large background noise. The dataset tested in this study contains different signal components, including: marine mammal signals^32^, propeller signals^33^ and background noise. Based on those analyses, the paper’s layout is divided as follows: Part 2 presents literature review about biotic signal structure, time-frequency domain approaches, the current limitations and proposed solution; Part 3 proposes solution of Cubic-splines interpolation combined with probability distribution in hidden space of SNN triple loss structure and compares the classification results with another published results in the same acutal dataset; and finally Part 4 presents the conclusion and future research directions.

## Literature review

### Marine mammal signal structure

Similar to humans’, marine mammals’ acoustic communication signals are produced by a set of tissues located in the larynx in the throat^32^. The larynx contains folds called vocal cords, and vibrations created by the airflow from the lungs into the mouth, depending on the shape and tension, can produce different sounds. All marine mammals produce sounds, and almost all sounds created by mammals are the result of the motion of air through various tissues.

In the first stage, air is pushed up from the lungs, creating pressure on the larynx, which opens to allow the air flow through; when the pressure decreases, the larynx automatically close. This closure again increases the pressure, and this process repeats^34^. The repeated opening and closing of the vocal cords generate sound wave frequencies, and these frequencies contain unique characteristics of each species. The shape and tension of the vocal cords can also be individually adjusted within each species to produce different sounds. In addition, the sound is also influenced by changes in the shape of the mouth, tongue, and lips.

Some marine mammals create different acoustic signals by slapping body parts onto the water surface. Among them, bottle-nose dolphins and humpback whales slap their tails on the water surface, creating broadband signals in the range of 30–12,000Hz^35^. The process of these mammals leaping out of the water and slapping body parts on the surface creates noise and also generates water bubbles. These bubbles burst and create acoustic pulses that propagate in the water, similar to the phenomenon of a propeller ship’s movement. In nature, marine mammals listen to these sounds to make critical decisions based on the specific characteristics of each species. Undoubtedly, understanding the mechanism of “hearing” is more important than “speaking” such as in human language processing^36^.

### Time-Frequency domain approaches

In the field of ocean acoustics, a underwater signal is defined as a unidirectional signal whose amplitude oscillates over time. The primary features of biotic and abiotic acoustic data are best collected within the frequency domain^37^, therefore techniques such as Fourier, Wavelet, and Mel transformations, which are time-frequency domain techniques, are the most useful solutions currently available for extracting information from underwater signals.

#### STFT

The classical Fourier transform is efficient with stationary signals, since they contain stable frequency components from the beginning to the end. The Fourier transform of the signal $s(\tau )$ is then written as an integral:

1 $$\begin{aligned} {F(w)=\int _{-\infty }^{\infty }exp(iw\tau )s(\tau )d\tau } \end{aligned}$$

where *t* is the time axis of the signal and *w* is the single frequency parameter.

The result of the integral from negative infinity to positive infinity over the time axis of the Fourier transform (FT) formula gives the frequency information of the signal but does not specify when that frequency information exists. That is, no matter where the frequency information appears on the time axis, it will give the same integration result. Therefore, if the signal is non-stationary, the FT will lose the characteristics of the signal. Then we use another FT transformation called the Short-time Fourier transform (STFT). STFT can be described as the following equation^38^:

2 $$\begin{aligned} {S(w,t_0)=\int _{-\infty }^{\infty }w(t_0-\tau )exp(iw\tau )s(\tau )d\tau } \end{aligned}$$

where *w*(*t*) is the window function and $(w,t_0)$ is the time-frequency coordinates of the base function.

The solution to overcome the disadvantage of FT used in STFT is: to multiply each segment of the signal by a window function. The window function has the same length as the segment of the non-stationary signal, which is small enough to satisfy the stability of the stationary signal^39,40^, and^41^. The DFT’s window is computed starting at t0; the window is applied to every signal segment, as the result of a window shift from 20% to 80% of the frame length. If the weight of the window function is equal to 1, the window is rectangular, that is, the response of the multiplication will be equal to the signal. The appropriate selection of the window will induce the stability of the frequency components during the Fourier transform of the signal in the time domain. The optimal window function will result in a narrow degree of main lobe and a low degree of lateral lobe, but when the degree of main lobe is too narrow, the degree of lateral lobe will increase. Therefore, for complex signals such as underwater signals, it is relatively difficult to optimize the window function. Wavelet transform can be combined to overcome this shortcoming.

#### Wavelet

The Wavelet transform (WT) is capable of handling linear and non-stationary signals, and thus, is capable of processing underwater signals in real-world conditions. With the STFT, the smaller the size of the window, the more we know about when the frequency has occurred in the signal, but the less information about the frequency itself. Vice versa, the larger the size of the window, the more we know about the frequency value and the less we know about the time. WT has high resolution in both the frequency and time domains. WT does not only show which frequencies are presented in a signal, but also show the time interval when such frequencies occur. This is done by using different sized windows. Instead of harmonic orthogonal functions, we use frames containing shift and compression functions in the frequency and time domains^42–44^, and^45^.

There are two distinct types of wavelet transform: continuous wavelet transform (CWT) and discrete wavelet transform (DWT). Among them, continuous wavelet transform is more suitable for feature extraction purpose. The continuous wavelet transform of a function *x*(*t*) is defined as the integral transform:

3 $$\begin{aligned} CWT(\lambda , \tau ) = \int _{-\infty }^{\infty }x(t) { \Psi _ {\lambda ,\tau } ^*} (t)dt \end{aligned}$$

where $\lambda$ is the scale parameter, $\tau$ is the positional parameter on the time axis and ${\Psi _{\lambda ,\tau } ^*}$ is the complex conjugate of ${\Psi _{\lambda ,\tau }}$, and ${\Psi _{\lambda ,\tau }}$ is the mother wavelet. Changing the value of $\tau$ can cause an expansion ($\lambda > 1$) or contraction $\lambda < 1$ on ${\Psi _{\lambda ,\tau }}$, and changing $\tau$ can shift the function *x*(*t*) along the time axis. As the $\lambda$ scale decreases, the wavelet becomes more compressed and takes into account only the short-time behavior of *x*(*t*); as the $\lambda$ scale increases, the wavelet becomes more stretched and considers the behavior of *x*(*t*) over a larger time increment. Thus, the wavelet transform provides a flexible time-scale window, which can be small for analyzing small-scale objects, or large for analyzing large-scale objects.

Similar to the STFT, the CWT scalogram is defined as the squared magnitude of the complex coefficients $CWT(\lambda , \tau )$ and it is a measure of the energy of the signal in the scale-time plane^46^. Scalogram represents the characteristics of a process in the scale-time plane; easily extracted multi-structures and temporary locations are the advantages of this representation method. The Haar wavelet using in this paper is a continuous function defined as^47^

4 $$\begin{aligned} \Psi (t)= \left\{ \begin{array}{lll} 1 &{}\text {if} &{} 0 \leqslant t< \frac{1}{2} \\ -1 &{}\text {if} &{} \frac{1}{2} \leqslant t < 1 \\ 0 &{}\text {if} &{} otherwise \end{array} \right. \end{aligned}$$

Wavelet Haar is a symmetric function which is easy to calculate and invertible without the edge effects that are the drawbacks of other wavelet methods. Wavelet Haar uses a rectangular window to sample a given time series. The window width is doubled at each step until the window covers the whole time series. Each pass of the time series generates a new time series and a new set of coefficients. The new time series is the average of the previous time series in the sampling window. Therefore, the coefficients represent the average change in the sample window.

The resolution of the wavelet obeys the Heisenberg’s uncertainty principle. The uncertainty principle states that it is not possible to know the momentum and position of a moving particle simultaneously, so it is not possible to determine together the time information and the frequency information at the same time. In the wavelet transform, higher frequencies give better resolution in the time domain, and lower frequencies give better resolution in the frequency domain, i.e., high frequencies can be detected more accurately than low frequencies. Therefore, we need to add a high resolution processing solution for low frequencies to increase the accuracy of the model.

#### Mel

Another typical methods for biomimetic filters used in digital signal processing are Mel frequency transform and Mel Frequency Cepstral Coefficients (MFCC). With the idea of recreating the human cognitive system, where the human ear structure being a linear spatial filter with low frequencies and a logarithmic space with high frequencies, these approaches in processing marine mammal signals are normally suitable. With a linear frequency axis, the Mel frequencies are calculated by the formula:

5 $$\begin{aligned} mel(f) = 2595log\left( 1 + \frac{f}{700}\right) \end{aligned}$$

Mel spectrograms are typically generated by dividing the signal into discrete time frames and then computing the frequency representation in the Mel-frequency scale for each frame^48^. So that, Mel frequency transform represents the frequency content of the underwater signal as the energy level of frequencies in the Mel-frequency scale for each time frame. Otherwise, MFCC^49^ uses the Mel spectrogram as a pre-processing step, followed by a cepstral transform to extract features from the signal. MFCC represents features of the audio signal as cepstral coefficients of the signal after applying Mel spectrogram pre-processing^50^. Thus, the advantage of Mel transform is to provide a clear representation of the underwater signal’s frequency content, making it suitable for detecting frequency structures in frequency domain. Therefore, the choice between Mel spectrogram or MFCC depends on the specific application; and Mel spectrogram is normally more appropriate for sound analysis.

### Limitations of Time–Frequency approaches

Based on the above analyses, the general limitations of time-frequency approaches based on passive sonar principle can be summarized into three main points.

#### The first

Underwater acoustic signals come from various sources such as artificial, biological, and environmental factors, and therefore vary in structure, size, intensity, and frequency distribution. In reality, the recorded signals are subject to frequency overlap between different sound sources.

#### The second

: The construction of models, data collection instruments, and datasets for underwater acoustics are not publicly available. Environmental signals typically do not have any specific frequency dominance over other frequencies, whereas artificial signals, which are a combination of wide-band and narrow-band signals, often have specific characteristic frequencies that are advantageous for detection.

#### The third

The stability of a dataset is dependent on multiple parameters such as environmental conditions, system deployment time, and hardware structure stability. During signal processing, parameters such as sliding window length and filter coefficients may lead to inaccurate results due to differences in the resolution of each operation.

Besides, studies analyzed^51–54^ have shown that deep models are effective but not always more accurate than complicated models. Therefore it is important to use a reasonable artificial intelligence model for certain classification tasks.

In conclusion, this paper presents a two-stage approach aimed at addressing the complexity of frequency and the limited amount of collected data before using an Siamese triple loss-Variation Auto Encoder model to enhance the quality of classification for underwater acoustic signals in a real environment.

### Solution approach

There are many different ways to present acoustic signals such as time series, frequency domain, time domain, or time-frequency domain. The choice of presentation method will determine the effectiveness of the classification model. The characteristics of marine communication signals are time-varying with complex rules; however, many marine animals have similar pronunciation structure to human’s sound tube^32^. Therefore, it is reasonable to use speech processing methods for marine communication signals. Besides, the short-time Fourier transform is relatively effective when dealing with periodic signals^55^ mixed with complex noise, while the wavelet transform is well adapted to the continuously changing signal environment^56^ such as shallow water. In practice, the process of collecting and storing underwater acoustic signals from acoustic sources requires a large number of parameters related to environmental conditions, system deployment timing, and hardware stability. Therefore, the stability of a datasets may be uncertain^37,57^. Additionally, when processing raw data using digital signal processing methods that utilize parameters such as window length and filter coefficients, classification errors can occur due to differences in the resolution of each operation. To mitigate these effects, an interpolation algorithm is used to enhance the quality of the data after transformation from raw acoustic data, before being fed into the classification modeling. Therefor, the paper uses two approaches to overcome the above limitations:

*Approach-1*: Fix data quality limitations

- Interpolations do not change the correlation structure of the data.
- Estimates the pixel values between known values, adding association between feature frequency components.

The effectiveness of Approach-1 is validated by applying the CSI algorithm to the STFT, and the resulting spectrograms are trained and evaluated by two published CNN model^38,58^ to compare the classification accuracy in the same actual dataset NOAA.

*Approach-2*: Fix frequency complexity

- Characteristics of marine communication signals are time-varying with complex rules.
- Many sea creatures have acoustic tubes like humans, it makes sense to use speech processing methods like Mel.
- The STFT transform is effective when dealing with cyclic signals that are mixed in a complex noise background.
- Wavelet transform is well adapted to continuously changing signal environments.

Therefore, to take advantage of each signal processing method, the records of actual dataset are pre-processed using STFT-CSI, wavelet, and Mel-frequency methods to generate characteristic spectrograms. In Approach-2, the spectrograms are independently evaluated in three separate cases as follows:

- The first case: evaluates the effectiveness of each STFT, wavelet, and Mel-frequency method when classified by the Rep-VGG-A0 model.
- The second case: evaluates the effectiveness of the first case with and without CSI using the Rep-VGG-A0 network model.
- The third case: evaluates the effectiveness of the second case when changing the classification model from Rep-VGG-A0 to SNN-VAE.

**Figure 1:**
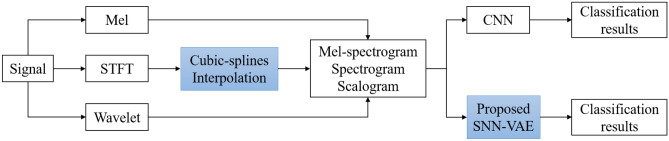
General proposed model for classifying marine mammal signals.

In conclusion, the general proposed model is described in Fig. 1.

STFT is a time-frequency analysis technique that uses sliding windows to compute the Fourier transform of a signal. However, one of the main disadvantages of STFT is that it assumes the frequency content of the signal is fixed in each time window, which is not always the case for non-stationary audio signals. STFT also suffers from a trade-off between frequency and temporal resolution, where increasing the window size for better frequency resolution results in poor temporal resolution.

The Mel frequency transform is based on the Mel scale, which is an approximation of the pitch perception of the human auditory system. Specifically, the logarithmic nature of the Mel-scale makes MFCC less sensitive to high-frequency components, which can limit its ability to capture important signal features. However, one of the main disadvantages of Mel is Mel is sensitive to noise and marine environmental conditions, which can affect the accuracy of the extracted features.

Wavelet transform is a time-frequency analysis technique that uses wavelets to represent signals at different scales and frequencies. One of the main disadvantages of the wavelet transform is that it requires a priori knowledge of the characteristics of the signal, such as its frequency and scale components. This can make it difficult to apply to underwater signals with unknown characteristics or complex spectral composition.

By analyzing the pros and cons of STFT, Mel, and Wavelet transforms when processing actual signals (especially underwater acoustic signals with overlapping characteristic frequency bands between different species), we proposed using spectrograms generated from each transform in a stacking spectrogram to optimize the benefits of each transform and overcome their respective drawbacks, which will help the pre-process to extract the complete features of underwater acoustic data better.

Spectral images (Mel-spectrogram, spectrogram, and scalogram) generated from Mel, STFT-CSI and Wavelet transform are converted into Red, Green, and Blue images, respectively. A RGB-spectrogram is represented by a three-dimensional matrix, with each dimension representing one of the three color channels: red, green, and blue. This paper combine the three spectral images by stacking them on top of each other to generate an RGB-spectrogram from separate red, green, and blue images. By concatenating the red, green, and blue images along the third dimension, which corresponds to the color channels, the result is an RGB-spectrogram with the same dimensions as the original red, green, and blue images. This set of RGB-spectrogram is the input for the Branch-1 and Branch-2 which are show in Fig. 1.

**Figure 2:**
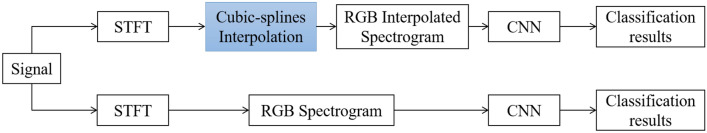
Evaluation model of interpolation algorithm on STFT.

Besides, the article verify the efficiency of the interpolation algorithm by using only RGB spectrogram generated from STFT as shown in Fig. 2.

**Figure 3:**
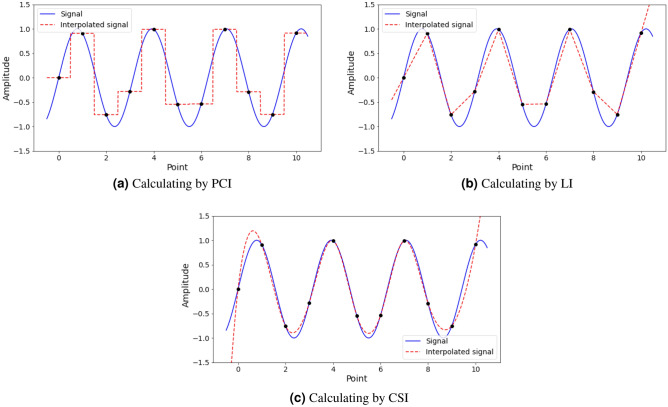
Calculating intermediate values by PCI, LI, and CSI.

Within the limited scope of this study, the paper is currently only purposing the difference when processing signals by interpolated signal with STFT. The main reason is that some limitations of Mel transform and Wavelet transform make the process of using higher order interpolation very complicated (Fig. 3).

With Mel frequency transform:

- Limited time resolution: The Mel frequency transform is a time-invariant transform, meaning that it provides a fixed frequency resolution over time. This can make it difficult to analyze signals that have rapidly changing frequencies or transient events.
- Limited frequency resolution: The Mel frequency transform may not provide enough resolution for underwater signal analysis in case of distinguishing between two closely spaced frequencies, the Mel scale may not be sufficient.

With Wavelet transform:

- Complexity: Types of Wavelet transforms are quite complex, with many different wavelets to choose from and a wide range of analysis parameters to set. It is very difficult to construct a general interpolation algorithm for the wavelet transform.
- Over-completeness: Wavelet transforms produce more coefficients than there are samples in the original signal. This can make the results difficult to interpret and may require additional processing steps to reduce the number of coefficients.

## Proposed solution of Cubic-splines interpolation combined with probability distribution in hidden space

### Proposal of the Cubic-splines interpolation pre-processing

#### Comparing result of interpolated signals

For a given sequence of data points, the goal of an interpolation algorithm is to provide intermediate data points to adjust the sequence to get specific requirements. Consider a function $\gamma :{\mathbb {R}}\rightarrow {\mathbb {R}}$, which maps a parameter $t\in {\mathbb {R}}$ to a value $\gamma (t)\in {\mathbb {R}}$. Assuming that the values of $\gamma (t_n)$ are discrete with $t\in {\mathbb {R}}$ and $n\in {\mathbb {Z}}$, interpolation algorithms estimate the value of $\gamma ^*(t)$ from the known values of $\gamma (t_n)$ with $t\in {\mathbb {R}}$, such that:

6 $$\begin{aligned} \gamma ^*(t) \approx \gamma (t) \end{aligned}$$

In practice, underwater signals may contain signals from one or more objects in the same record. Therefore, it is necessary to evaluate and compare the quality of each interpolation method.

- If the simplest interpolation operation, Piece-wise constant interpolation (PCI), is used with a parameter $t\in {\mathbb {R}}$, the nearest parameter $t_n$ and the value of the interpolation function is determined by the formula:
  7 $$\begin{aligned} \gamma ^*(t)=\gamma (t_n) \end{aligned}$$
- If linear interpolation (LI) is used with a parameter $t_i$ in the range of $t_{n-1}$ to $t_n$, the value of the interpolation function is determined by the formula:
  8 $$\begin{aligned} \gamma ^*(t)=\gamma (t_{n-1}) + (\gamma (t_{n})-\gamma (t_{n-1}))\frac{t-t_{n-1}}{t_{n} - t_{n-1}} \end{aligned}$$
- If Cubic-splines interpolation (CSI) is used with a parameter $t_i$ in the range of $t_{n-1}$ to $t_n$, the value of the CSI function is calculated using formula (9):
  9 $$\begin{aligned} \gamma ^*(t) = a_i(x-x_i)^3 + b_i(x-x_i)^2 + c_i(x-x_i) + d \end{aligned}$$

**Figure 4:**
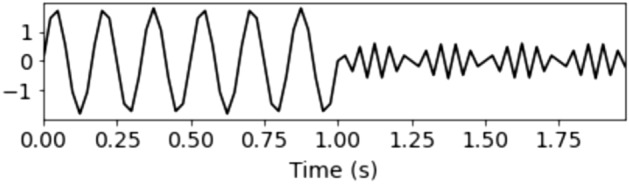
Signal consisting of two waves.

Apply formulas (7), (8), (9) for the two following cases **A** and case **B**:

**A.** In the case where the underwater data sequence contains only one sinusoidal wave, assuming that it has the form $\gamma (t)=sin(2t_n)$ with $t_n\in [0,11]$, the intermediate values are calculated using the PCI, LI, and CSI are represented respectively as follows in Fig. 3a–c:

**B.** In the case where the underwater data sequence contains more than one sinusoidal mechanical wave, assuming that it has the form $\gamma _1(t)=1.8sin(2\pi 6t)$ and $\gamma _2(t)=0.6sin(2\pi 18t)$, the intermediate values are calculated using the same interpolation methods as in Case A:

The signal consists of two waves $\gamma _1(t)$ and $\gamma _2(t)$, as described in Fig. 4.

**Figure 5:**
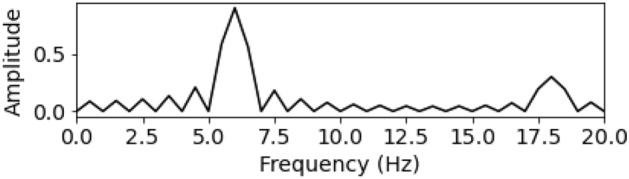
Spectrum of consistent signal.

The signal after using FFT analysis is described in Fig. 5.

**Figure 6:**
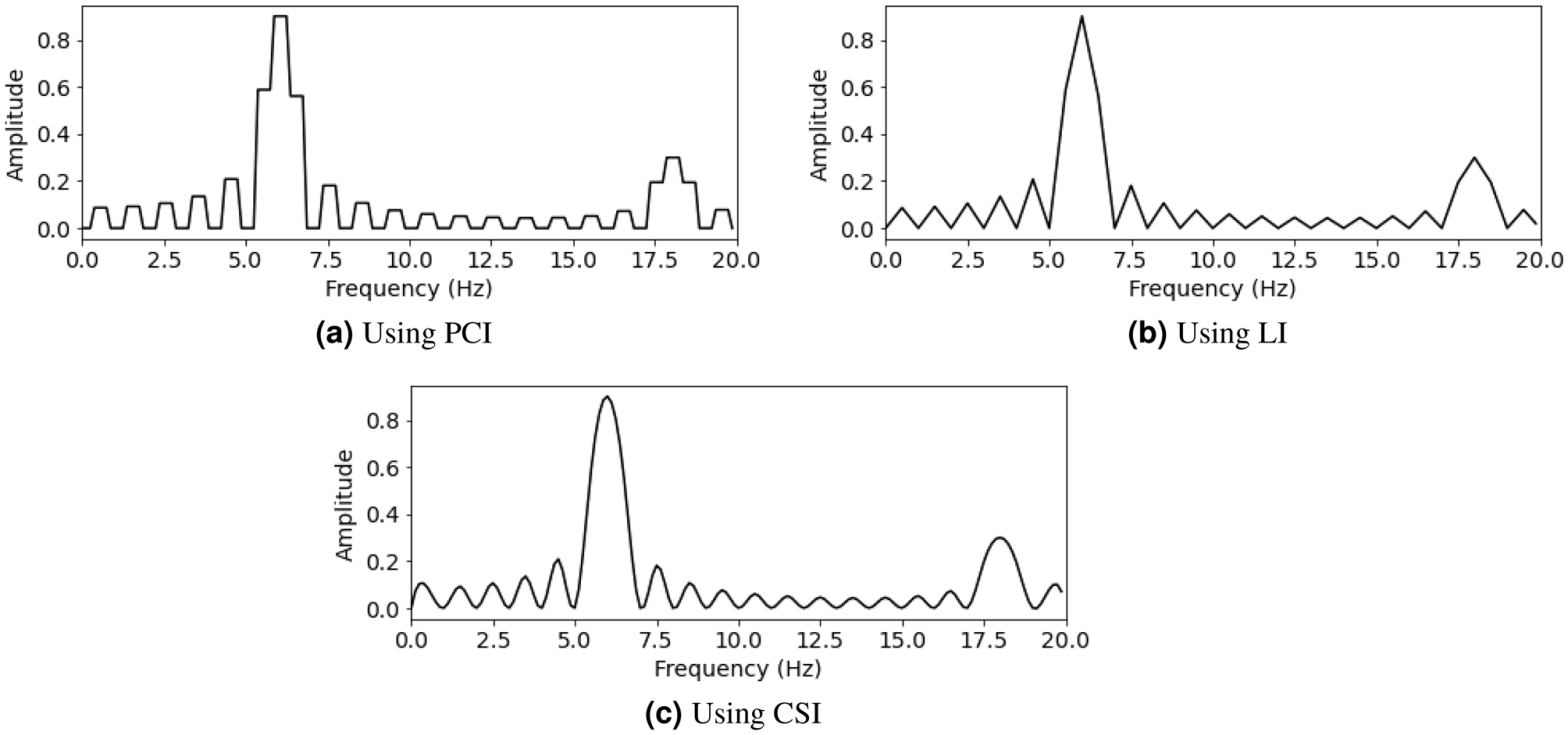
Spectrum after using PCI, LI, and CSI.

The spectral values after being processed by the PCI, LI, CSI methods are described in Fig. 6a–c, seperately as follows:

The values of the PCI method are discrete points (Fig. 3a and Fig. 6a) indicating that there will be more limitations than LI method (Fig. 3b, Fig. 6b) and CSI (Fig. 3c and Fig. 6c) method, if PCI is applied to nonlinear functions which are popular in practice. Piece-wise constant interpolation (PCI) involves dividing the signal into intervals and assigning a constant value to each interval. The value of each interval is determined by the value of the signal at the beginning of that interval. This method is simple and computationally efficient. However, it may result in inaccurate estimates of the original signal, particularly when the signal frequency components changes complicated.

Linear interpolation (LI) involves connecting two known points with a straight line to estimate the value of an unknown point. This method is more accurate than PCI, as it takes into account the slope of the signal between two known points. The accuracy of linear interpolation can be further improved by using higher-order polynomial interpolation methods such as Cubic spline interpolation (CSI). Cubic-spline interpolation involves the use of a cubic polynomial to estimate the value of a signal at a specific point. This method provides more accurate estimates of the original signal compared to piece-wise constant and linear interpolation. It also produces smoother interpolations compared to the other two methods. Thus, the choice of interpolation method depends on the specific needs of the application, including the trade-off between computational efficiency and accuracy.

#### Proposed interpolation algorithm

Cubic spline interpolation is a well-established method for approximating a function within a set of discrete data points. While cubic splines can be applied to both one-dimensional and two-dimensional data sets, there are differences between using cubic spline interpolation for one-dimensional (the result of Fourier transform) and two-dimensional (the result of the Short time Fourier transform) data, based on mathematical representation of CSI.

Assuming a Cubic-splines interpolation in the interval from $x_0$ to $x_n$ is defined by a set of polynomials$f_i(x)$, the mathematical representation of the Cubic-splines interpolation^59^ is:

10 $$\begin{aligned} f_i(x) = A_i(x-x_i)^3 + B_i(x-x_i)^2 + C_i(x-x_i) + D \end{aligned}$$

where *i* = 1,2,..., $n-1$; and *n* is the number of knots. Therefore, $n-1$ cubic polynomials will form the Cubic-splines interpolation. If we have $n+1$ knots $(x_0, y_0)$, $(x_1, y_1)$,..., $(x_n, y_n)$ with $x_{i+1}$ – $x_i$ = *q*, then the interpolation function $f_i(x)$ must satisfy four conditions simultaneously:

11 $$\begin{aligned} f_i(x)&= y_i \end{aligned}$$

12 $$\begin{aligned} f_i(x_{i+1})&= f_{i+1}(x_{i+1}) \end{aligned}$$

13 $$\begin{aligned} f'_i(x_{i+1})&= f''_{i+1}(x_{i+1}) \end{aligned}$$

14 $$\begin{aligned} f''_i(x_{i+1})&= f''_{i+1}(x_{i+1}) \end{aligned}$$

Solving formula (11), we have: $D=y_1$. Expanding the first and second derivatives of $f_i(x)$:

15 $$\begin{aligned} f'_i(x)&= 3a_i(x-x_i)^2 + 2b_i(x-x_i) \end{aligned}$$

16 $$\begin{aligned} f''_i(x)&= 6a_i(x-x_i) + 2b_i \end{aligned}$$

Let $M=f''_i(x)$, expanding formula (16):

17 $$\begin{aligned} M_i = f''_i(x_{i}) = 6a_i(x_i-x_i) + 2b_i = 2b_i \end{aligned}$$

Therefor: $b_i = \dfrac{M_i}{2}$. Continuing to expand formula (16):

$$\begin{aligned} f''_{i+1}(x_{i+1})&= 2b_{i+1} \\ f''_i(x_{i+1})&= 6a_i(x_{i+1}-x_i) + 2b_i = 6a_iq +2b_i \end{aligned}$$

Substituting this result into formula (14) to calculate the values:

$$\begin{aligned} 2b_{i+1}&= 6a_iq +2b_i \\ a_i&= \dfrac{2(b_{i+1}-b_i)}{6q} = \dfrac{M_{i+1}-M_i}{6q} \end{aligned}$$

Similarly, expanding (13) and (14) to calculate *c*:

$$\begin{aligned} c_i = \dfrac{y_{i+1}-y_i}{q} - \left( \dfrac{M_{i+1}+2M_i}{6}\right) h \end{aligned}$$

**Figure 7:**
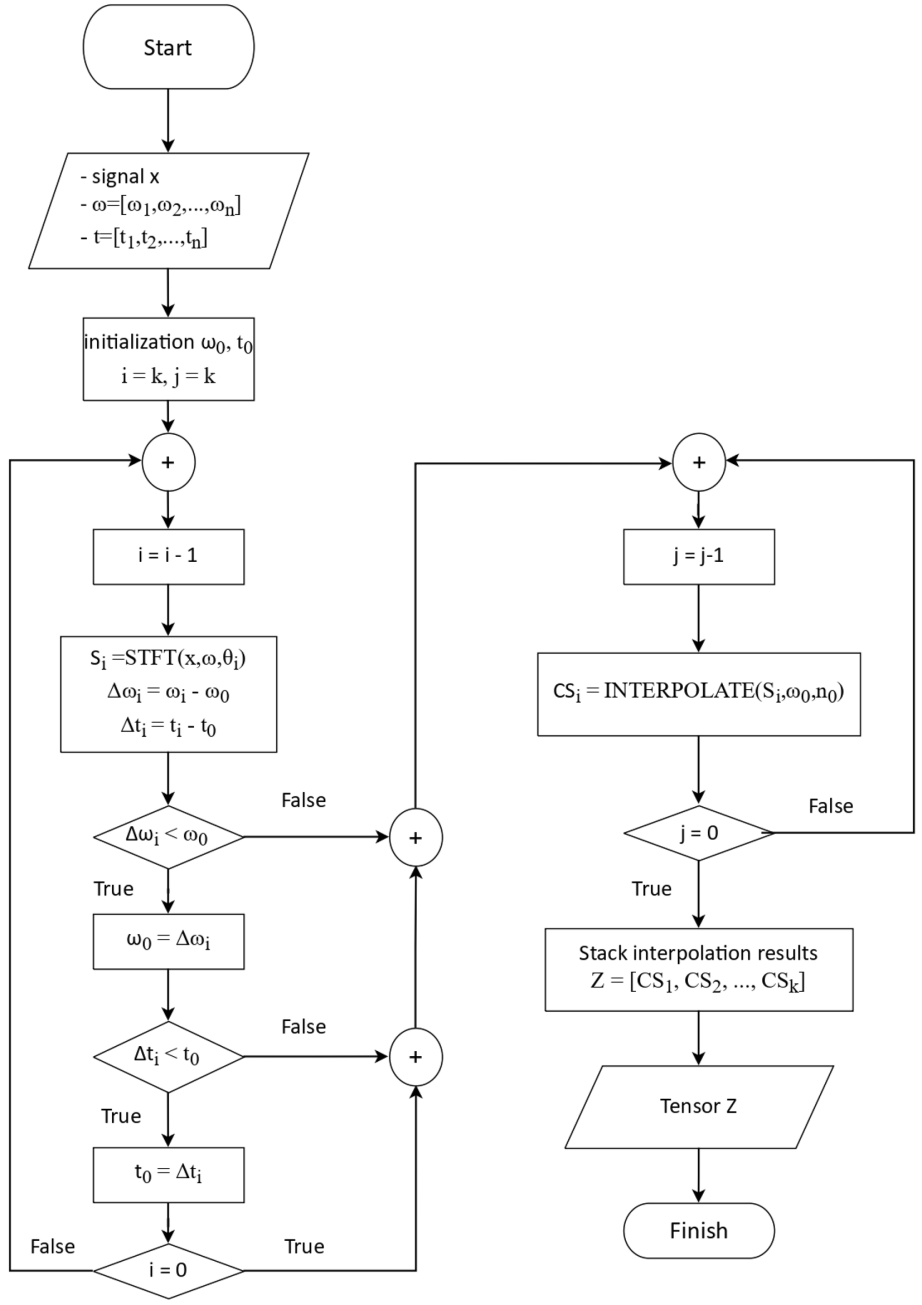
Flowchart of proposed algorithm for processing marine mammal signals.

The cubic-splines interpolation solution will avoid the instability of high-degree polynomial interpolation and the limitations of statistical models. Pre-processed data will be passed through a cubic-splines interpolation to estimate intermediate pixel values between known values, thereby improving the quality of features and creating a new spectrogram with coherence between frequency components. In one-dimensional data, cubic splines interpolate along a single axis, producing a curve that smoothly passes through the data points. The resulting curve is a series of connected cubic polynomial functions, each defined by the values at the two neighboring data points, and is continuous up to its second derivative. This continuity ensures that the resulting curve is smooth and aesthetically pleasing. In contrast, two-dimensional data such as spectrogram, requires a surface interpolation approach. Surface interpolation involves approximating a smooth surface that passes through the data points in two dimensions. The surface is represented by a series of connected cubic polynomial functions in both the time and frequency directions, allowing for smooth transitions between adjacent points in both dimensions. To perform cubic spline interpolation for two-dimensional data, the data points must first be organized into a grid or matrix format. A two-dimensional spline function can then be constructed by interpolating along each axis separately. This results in a piece-wise polynomial function that approximates the surface using cubic polynomial functions in both the x and y directions. The surface is then evaluated at any desired point by computing the values of the surrounding polynomial functions and interpolating between them. The resulting function is continuous up to its second derivative in both directions. The result of pre-processing is a 2D spectrogram in which the intensity on the spectrogram represents the strength of the signal. Each set of three spectrograms generated from the three transformations on the same signal samples are stacked together to form a final spectrogram. The proposed Cubic-splines for pre-processing and proposed algorithm flowchart are shown in Fig. 7 and Algorithm 1.

**Algorithm 1:**
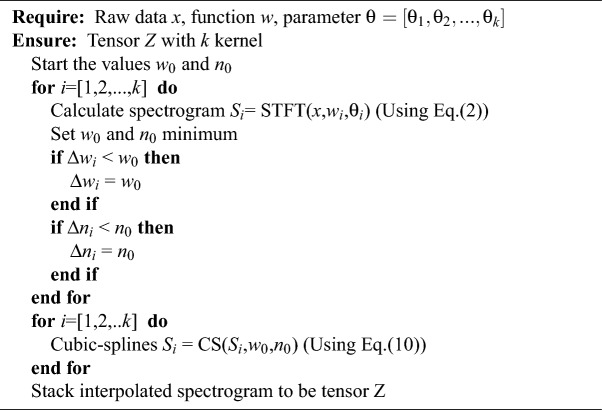
Proposed Cubic-splines interpolation for pre-processing

The proposed CSI algorithm on frequency domain performs interpolation the previously dismissed based on the calculated data. This represents in a new representation of sonar signals to further explore the relationship between the extracted characteristic frequencies.

### Proposal of probability distribution in the hidden space for Siamese triple loss network

#### Drawback auto-encoder

The Auto-Encoder (AE) model consists of three main parts:

- The Encoder will take the input features, remove the unnecessary ones, and then compress the selected features into a feature vector with fewer dimensions than the original. The result of this process is a smaller space that can hold all of the input features. In this study, with an input image of 128x128x3 RGB color channel, the use of the encoder is necessary.
- Latent space: This bottleneck area contains feature vectors with important information that has been compressed from the input. Hence:
  - The dimension of the bottelneck is smaller than the input dimension
  - The smaller the bottleneck, the less overfitting because the model will have to select the more important information to carry, and thus the capability to contain unnecessary features is reduced. However, if the Bottelneck is too small, then too little information can be stored, reducing the ability to decode of the Decoder. Therefore, this area should be kept at a balanced level.
- The Decoder will decode from the Latent space to try to generate a new spectrogram that has the closest relationship to the old image.

However, even if the data dimension after being encoded (latent dimension) is low, the Auto-Encoder model can still lead to overfitting, because AE focuses on the sole goal of reducing the loss as much as possible. As a result, the latent space of AE will encounter two problems:

- Loss of continuum: close points in latent space can provide very different decoded data
- Loss of completeness: Some points of latent space may provide meaningless content once decoded.

Therefore, instead of encoding the input as a single point, this paper encodes it as a distribution over the latent space, and then normalizes that distribution using a VAE model.

#### Variational auto-encoder

**Figure 8:**
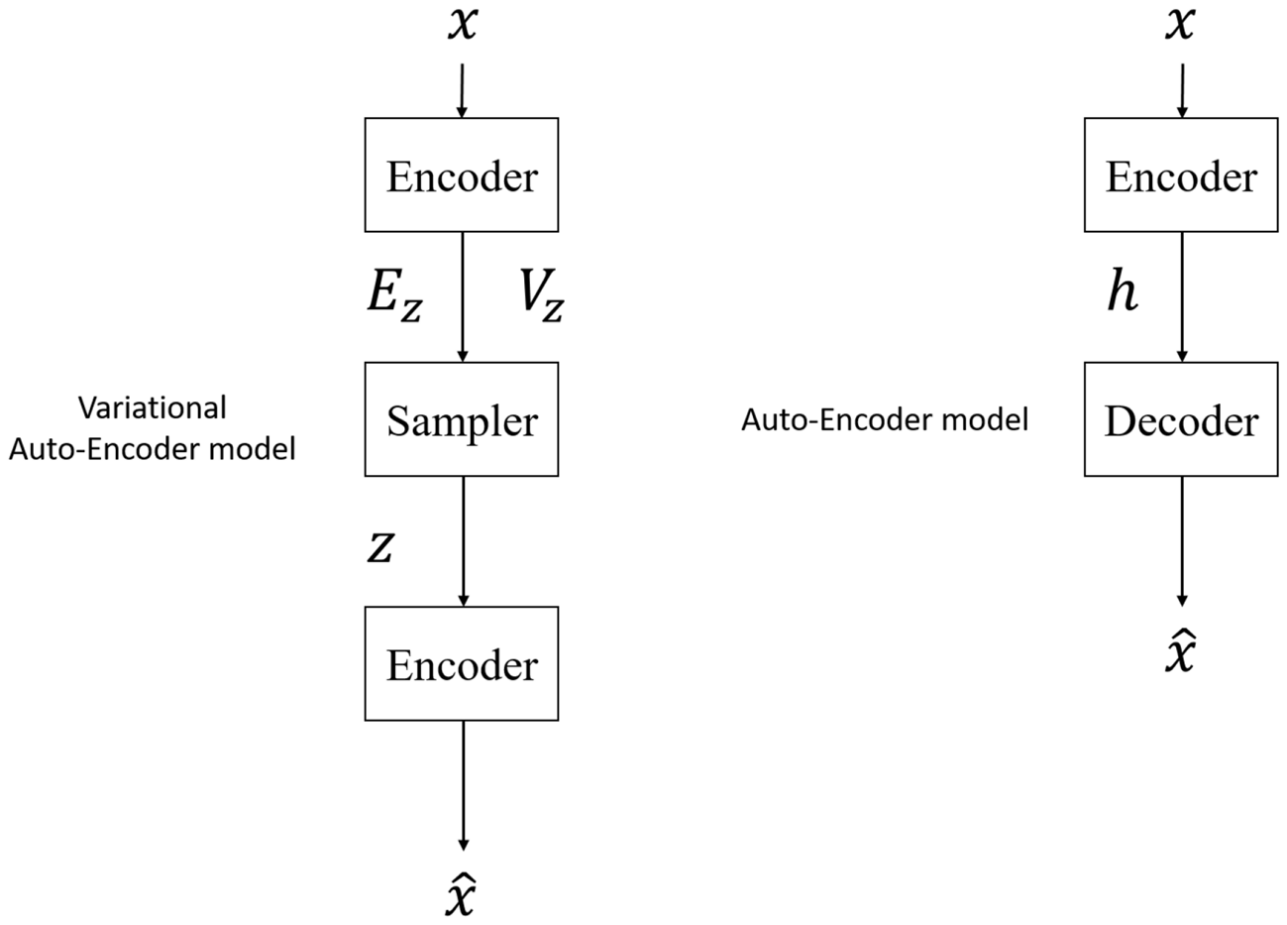
Comparing Auto-Encoder and Variational Auto-Encoder.

For the Auto-Encoder model: The encoder will map the input *x* to a hidden vector *h* (usually with dimensions less than *x*) called a code. The hidden vector *h* is then transformed by the decoder into the output of the ${\hat{x}}$ model. The output is then used to compute the loss function. Figure 8 shows the comparison approach between Autoencoder and Variational Auto-Encoder model.

Meanwhile, for the Variational Auto-Encoder (VAE) model, instead of mapping to a hidden vector *h*, the VAE code consists of two vectors ${\mathbb {E}}(z)$ and ${\mathbb {V}}(z)$, where *z* is a random variable distributed normally *d* with mean vector ${\mathbb {E}}(z)$ and variance vector ${\mathbb {V}}(z)$. The encoder will be a map $f: R^{d_x} \mapsto R^{2d_h}$ (VAE’s *h* will be a vector concatted by 2 vectors ${\mathbb {E}}(z)$ and ${\mathbb {V}}(z)$). From two vectors ${\mathbb {E}}(z)$ and ${\mathbb {V}}(z)$, a hidden vector *z* will be sampled from a normal distribution with corresponding mean and variance. The vector *z* will then be transformed by the decoder into ${{\hat{x}}}$. Instead of mapping the input *x* to a single point in the latent space as in the autoencoder, VAE maps *x* to a probability distribution from which a sample *z* will pass through the decoder. Therefore, the latent space of VAE is a smooth and continuous space, which limits the impact on data recovery at the output.

The loss function of VAE includes two components: reconstruction loss and regularization loss:

18 $$\begin{aligned} {\mathscr {L}}(x, {{\hat{x}}}) = {\mathscr {L}}_{reconstruct} + \beta {\textbf{K}}{\textbf{L}}(z, N(0, I_d)) \end{aligned}$$

- Reconstruction loss is the amount of information lost after reconstruction, similar to the case of AE.
- Regulization loss measures the distance between the normal distribution with the mean ${\mathbb {E}}(z)$ and the variance ${\mathbb {V}}(z)$ for the normal normal distribution *d* dimension $N(0, I_d)$ (which is the distance between 2 probability distributions). Regulization loss manages the regularity of the latent space, expressed as Kulback-Leibler divergence (KL divergence). This measure calculates the difference between 2 probability distributions and reaches zero when the two distributions are considered to be the same. The loss function will try to minimize the KL divergence between the original distribution and the parameterized distribution, thereby preventing the network from learning narrow distributions and trying to bring the distribution closer to the unit normal distribution.

19 $$\begin{aligned} \text{ loss }=\Vert x-x{\hat{x}}\Vert ^{2}+\textrm{KL}\left[ N\left( \mu _{x}, \sigma _{x}\right) , N(0, I)\right] \end{aligned}$$

**Figure 9:**
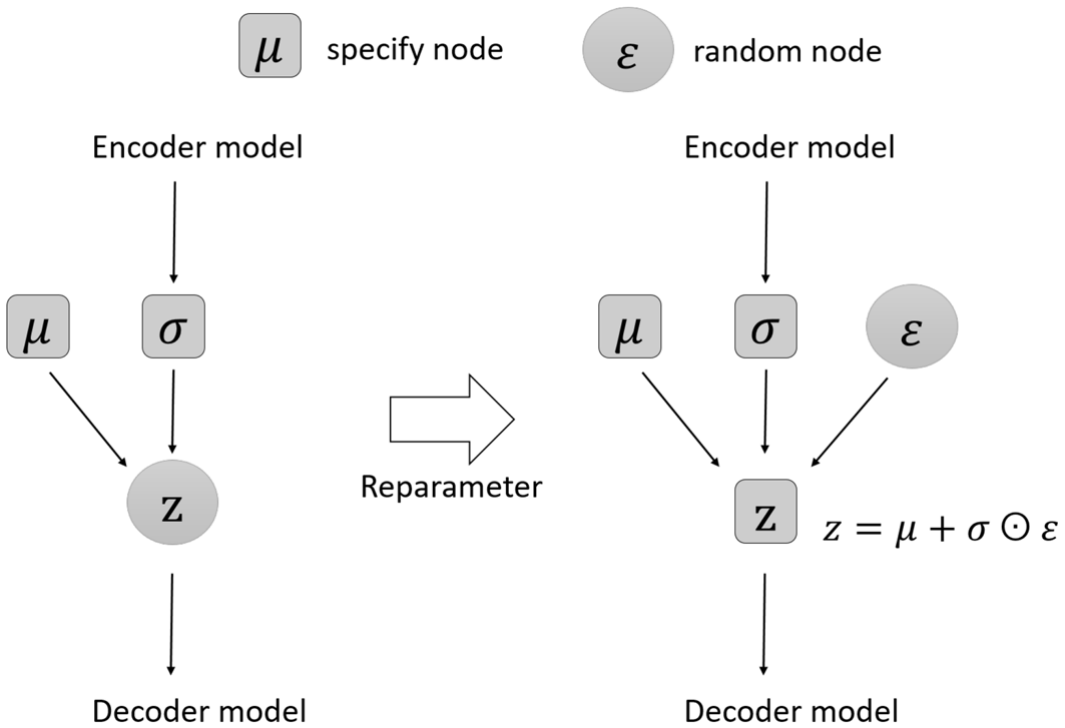
Reparameter.

To train neural networks, gradient descent algorithm is normally used, that is, derivative is performed. In VAE, we need to re-parameterize the hidden vector *z* as shown in Fig. 9. If *z* is a random variable following a Guassian distribution with mean $\mu _x$ and covariance $\sigma ^2$, then we can expand *z* as follows:

20 $$\begin{aligned} z = {\mathbb {E}}(z) + \varepsilon \odot \sqrt{{\mathbb {V}}(z)} \end{aligned}$$

with $$\varepsilon \sim N(0, I_d)$$.

Since ${\mathbb {E}}(z)$ and ${\mathbb {V}}(z)$ are the output of the encoder, the derivative can back-propagate to perform, from which, we have the formula:

21 $$\begin{aligned} {\textbf{K}}{\textbf{L}}(z, N(0, I_d))=\frac{1}{2}\sum _{i=1}^d( {\mathbb {V}}(z_i) - \log {\mathbb {V}}(z_i) - 1 + {\mathbb {E}}(z_i)^2) \end{aligned}$$

#### Siamese neural network

**Figure 10:**
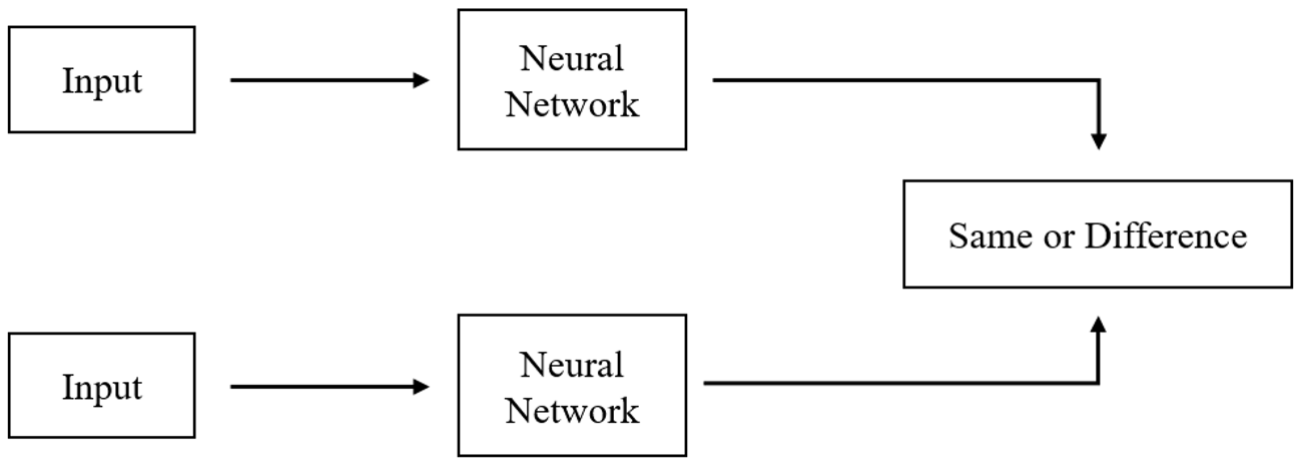
A typical Siamese Neural network.

Siamese Neural Network (SNN) as shown in Fig. 10^60^ is a neural network architecture containing two or more identical sub-networks that have the same configuration with the same parameters and weights. Updates of parameters are reflected on all of its sub-networks simultaneously^61^. SNN is used to find the similarity of input data by comparing their feature vectors.

If new classes need to be added or removed, the neural network needed to be updated (trained) on the entire dataset (both new and old data). In addition, deep neural networks require a large amount of data to be trained. SNN^62^, however, learns to find similarities between Input Data, so it can classify new data classes without having to be retrained.

The operating model of SNN is as follows:

- Step 1: Select a pair of Input Data (being images in this paper) from the dataset.
- Step 2: Pass each image through each sub-network of the SNN for processing. The output of each sub-network is an Embedding vector.
- Step 3: Calculate the Euclidean distance between those two Embedding vectors.
- Step 4: A Sigmoid Function can be applied over the distance to provide a Score value in the interval [0,1], representing the similarity between the two Embedding vectors. The closer Score is to 1, the more similar the two vectors are and vice versa.

SNN also focuses on learning Features in deeper layers, where similar Features are placed close to eachother. Therefore, the model will understand the similarity in the characteristics of the inputs. Moreover, SNN is also capable of combining with other classifiers, because the learning mechanism of SNN is a Convolutional neural network with the output layer removed. In this study, a classification model that combines the Siamese triple loss network and the Variational Auto-Encoder is proposed to improve the classification results of sound sources with relatively similar characteristics such as marine mammal signals, propeller signals and background noise.

#### Rep-VGG neural network

Rep-VGG network is an improvement from the convolutional network VGG-16 in 2014; VGG-16 is the first architecture to change the order of blocks when stacking multiple convolution and max pooling layers instead of alternating one convolution with one max pooling layer, based on the view that deeper CNN can better extract features. The appearance of the multi-branch architecture GoogleNet in 2015^63^ and ResNet in 2017^64^ gave better results thanks to advantages such as: limiting derivation when performing backpropagation, reducing the dependency of the main model on a certain branch, and allowing the features of the former layer to be concatted and passed directly to the next layer to avoid information loss.

However, the multi-branch architecture results in a slow inference process, which consumes significantly more computer RAM than the single-branch architecture does. Therefore, the Rep-VGG model introduces multi-branch training yet single-branch inference by parametric reconstruction technique.

The Rep-VGG architecture and its variants^65^ all split into two separate parts, single-branch for inference and multi-branch for training. They are also divided into five phases, each of which consists of 1 or more blocks. All the first blocks of each stage use a stride of 2, while the remaining blocks use a stride of 1. Sliding windows have the size of [3x3], using the Relu activation function (the [1x1] convolution branch and validation branch are only used for training) and completely eliminating the pooling layer of the classical VGG structure. The multi-branch design along with the skip connection step will increase the complexity of the model, thereby enhance feature learning and avoid derivative annullation.

#### Proposed model Siamese triple loss variational auto-encoder

The two sub-networks with CNN structure in the SNN produce two encoding vectors, $x_1$ and $x_2$ representing the first and second spectral images, respectively. $x_1$ and $x_2$ have the same dimensions. The function *f*(*x*) has the same effect as a fully connected layer transformation in a neural network to create nonlinearity and reduce the data dimensions. When $x_1$, $x_2$ are the same object or not the same object, the value of $||f(\mathbf {x_1}) - f(\mathbf {x_2})||^{2}$ will be a small or large value, respectively.

**Figure 11:**
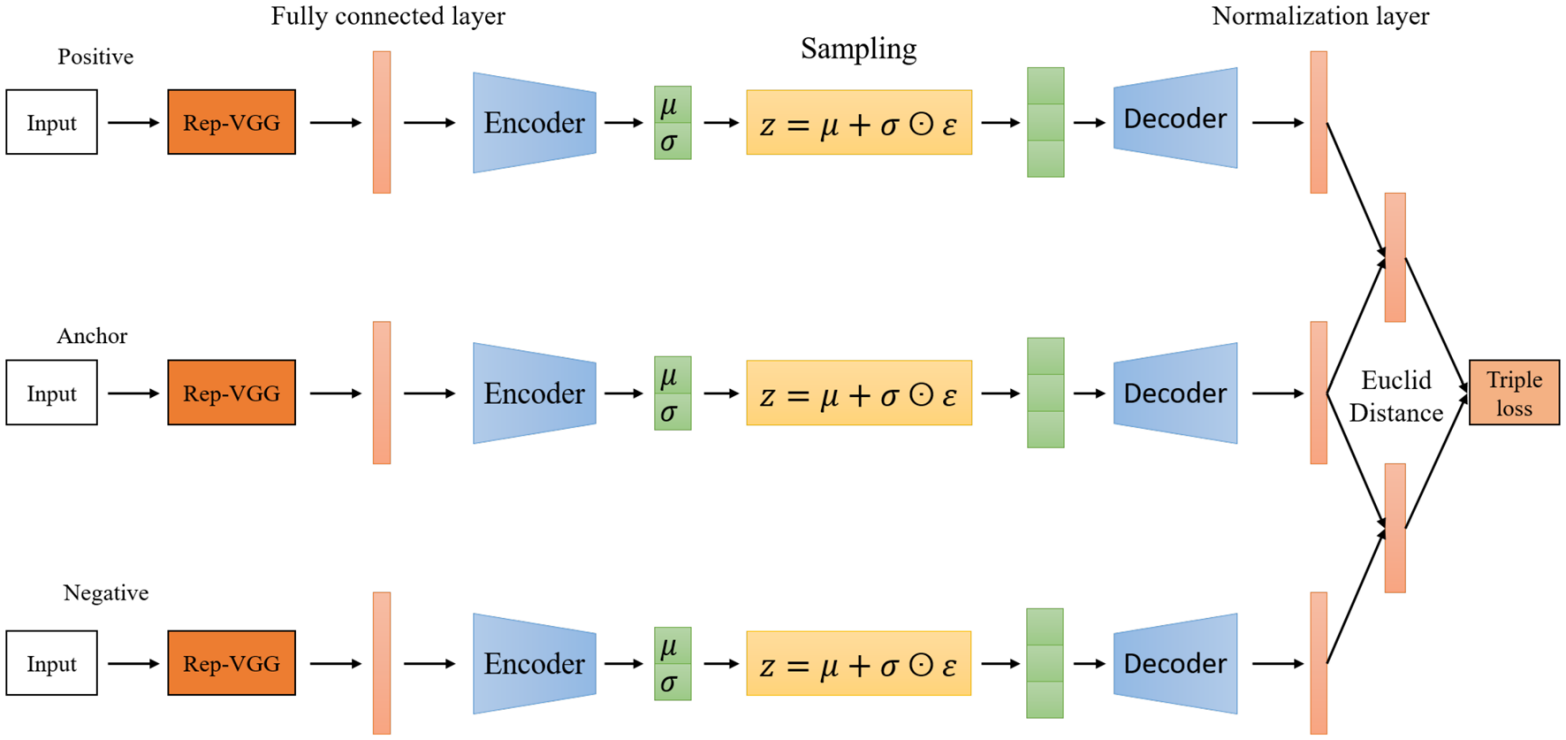
Triple loss with Variational AE.

The proposed diagram of the proposed model with CNN structure being Rep-VGG is described in Fig. 11.

Since, the sub-network applies a convolution neural network, the data dimensions are reduced to only 128. Therefore, the inference and prediction process is faster while at the same time the accuracy is guaranteed. The loss function used in this model is a triple loss function, which is capable of simultaneously learning the similarity between two spectrograms in the same class and distinguishing spectrograms that are not in the same class. The goal of the loss function is to minimize the distance between two images when they are negative and maximize the distance when they are positive. Therefore, we need to select sets of 3 images such that:

- The Anchor and Negative images are the most different: the distance $d(\textbf{A}, \textbf{P})$ needs to be large. Spectrograms of the same object recorded at different times are selected to form pairs. This selection will help the model learn better.
- The Anchor and Negative images are the most similar: the distance $d(\textbf{A}, \textbf{N})$ needs to be small. This is similar to distinguishing the spectral images of the marine mammal signal and the propeller signal with the same frequency band but different intensity and distribution.

Ultimately, the aim is to ensure that the training data follows the formula:

22 $$\begin{aligned} d(\textbf{A}, \textbf{P}) < d(\textbf{A}, \textbf{N}) \end{aligned}$$

whereby:

23 $$\begin{aligned} \left\| f\left( A \right) - f\left( P \right) \right\| ^{2} - \left\| f\left( A \right) -f\left( N \right) \right\| ^{2} \le 0 \end{aligned}$$

To prevent the neural network from outputting all equal values, leading to a constant output function *f* equal to zero, the model introduces a boundary value $\alpha$ to make *f* different from zero, but still close to zero.

24 $$\begin{aligned} \Vert f(A)-f(P) \Vert ^{2}- \Vert f(A)-f(N) \Vert ^{2}+ \alpha \le 0 \end{aligned}$$

The loss function at the output of the SNN network is defined with *n* set training:

25 $$\begin{aligned} {\mathscr {L}}(\textbf{A, P, N}) = \sum _{i=0}^{n}\max (||f(\textbf{A}_i)-f(\textbf{P}_i)||^{2} - ||f(\textbf{A}_i)-f(\mathbf {N_i})||^{2}+ \alpha , 0) \end{aligned}$$

where *n* is the number of the sets of 3 spectral images to be trained.

Choosing a random set of three spectral images can easily lead to the inequality (22) because the probability of similarity between random spectral images is low. That is, most of the cases will satisfy the inequality (22) and do not affect the value of the loss function because the value tends to reach 0. Therefore, learning Negative images that are too different from Anchor would not make much sense. Thus, we can use a semi-hard triplet where the Negative is not closer to the Anchor than the Positive, but there is still positive loss: $d(\textbf{A}, \textbf{P})< d(\textbf{A}, \textbf{N} ) < d(\textbf{A}, \textbf{N}) + \alpha$.

In each iteration of training, our final loss function for an input triplet is demonstrated by the formula:

26 $$\begin{aligned} {\mathscr {L}}_{SNN-VAE} = {\mathscr {L}}(\textbf{A, P, N}) + {\mathscr {L}}_{reconstruct} + \beta {\textbf{K}}{\textbf{L}}(z, N(0, I_d)) \end{aligned}$$

On each branch of the SNN-VAE model, when training, a block of Rep-VGG has three sub-branches consisting of a [3x3] convolution layer, a [1x1] convolution layer, and a validation branch. When the model starts learning, a Rep-VGG block has only one branch, being the [3x3] convolution layer. All the following layers are combined with the BN layer to pass the model’s parameters from training to inference. For normal model, the weight function after training is saved and cannot be activated for a model with a different architecture. However, by re-parameterizing the vector, Rep-VGG can convert the weight function from multi-branch to single-branch. The number of parameters of Rep-VGG is reduced compared to that of VGG and ResNet, thereby limit the computation volume of the model and reduce errors.

The efficiency of the single-branch model comes from the architecture that breaks down into sub-blocks. With $\delta$ sub-blocks trained, the model can consist of $2^\delta$ sub-models because each block is divided into 2 branches. This makes the multi-branch connection more comprehensive and not depend on any layer. If Rep-VGG has $\delta$ blocks, then the model will have $3^\delta$ sub-models; this helps Rep-VGG architecture better represent data.

## Experiment results

### Actual dataset (NOAA) of marine mammals signal

**Figure 12:**
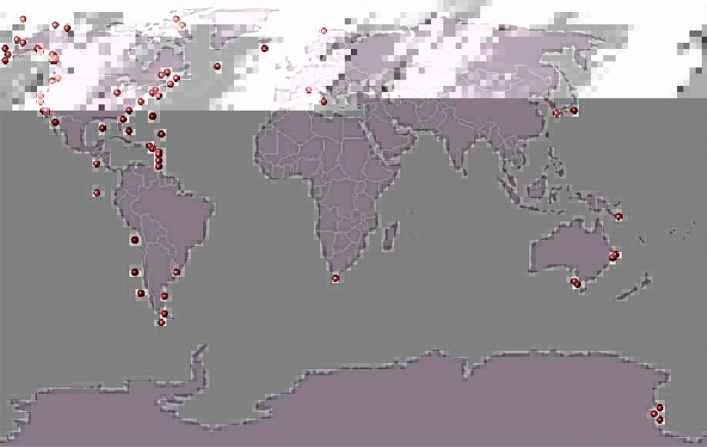
Map of NOAA data collection points.

The National Oceanic and Atmospheric Administration (NOAA) Marine Mammal Sound Database^66^ was established in 1991 with the aim of providing convenient access for scientists worldwide to recordings of marine mammal communication signals. The dataset is structured and regularly updated with specific indexing, and data retrieval is simple and intuitive from the website of the US-based Oceanographic Institute. The dataset has been digitized into wav audio files containing behaviors related to the sound production process of many marine species in their natural habitat. The recordings are diverse and include both biotic sounds of multiple species within a class and sounds from abiotic sources (NOAA data collection points are shown in Fig. 12)^67^.

Since the start of data collection in 1949 until 2022, the full NOAA dataset contains over 15,000 annotated recordings of 60 marine mammal species, including 1,694 high-quality, low-background noise recordings of the 32 most common species, known as the BOC-NOAA dataset. Table  1 presents four classes’s information of marine mammal in BOC-NOAA data.

**Table 1:** Distribution marine mammal classes in BOC-NOAA dataset.

	Common dolphin	Spinner dolphin	Humpback whale	Killer whale
Amount	52 (records)	114 (records)	64 (records)	35 (records)
Duration	658 (s)	338 (s)	831 (s)	94 (s)

Two types of dataset has been categorized by scientists by species and groups of species in small time durations ranging from 3 to 5 seconds per recording, which is stored as a wav file^68^. The NOAA provides a rich resource for studying changes in marine mammal communication processes related to changes in ocean noise levels spanning over seven decades^32^ and serves as a reference for classifying marine mammal signals in other regions worldwide.

### Probability of detection and classification indicators

The relation between the signal of interest and the remaining signals (considered noise) in the ocean determines whether an active or passive sonar system can detect a reflected or radiated signal from the target. To determine the presence of a signal, sonar systems set a detection threshold (DT) such that when the ratio of the signal of interest to the noise level at the receiver is higher than the DT, the system decides that there is a signal, and vice versa. Setting the DT too high reduces false alarms but increases the likelihood of missed signals, while setting it too low increases false alarms.

**Figure 13:**
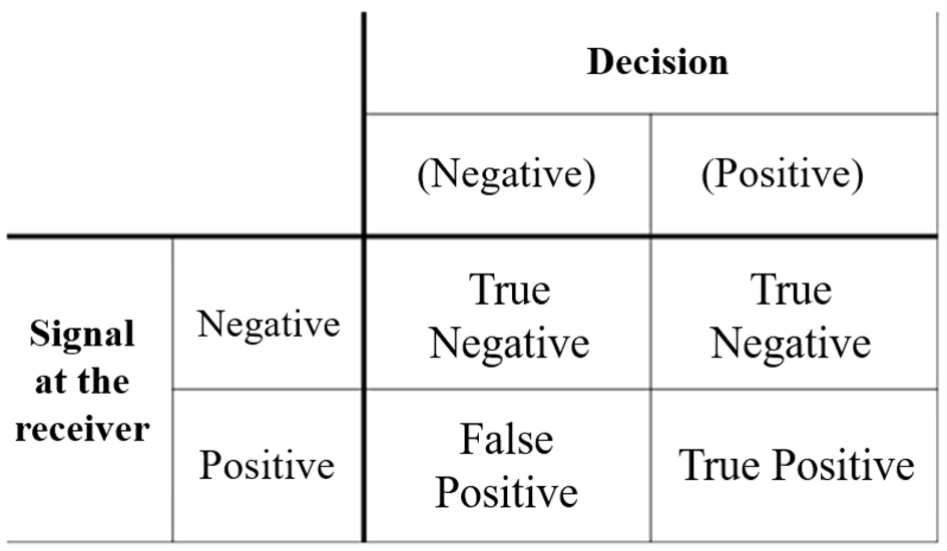
Four cases of signal detection probability.

According to the signal detection theory^69^, Fig. 13 illustrates four possible outcomes when deciding whether a target signal exists or not:

- The signal exists and a correct decision is made, referred to as true positive *TP*;
- The signal exists but an incorrect decision is made, referred to as false positive *FP*;
- The signal does not exist but an incorrect decision is made, referred to as false negative *FN*;
- The signal does not exist and a correct decision is made, referred to as true negative *TN*.

Figure 13 shows a confusion matrix with four different and distinct cases. Based on these indices, other metrics such as Accuracy, Precision, and Recall are calculated and used in specific scenarios to reflect the effectiveness and information of the classification system.

- Accuracy is a general metric that describes how well the model performs on all classes in the dataset and is often used when all classes are equally important. Accuracy is calculated as the ratio of the number of correct predictions to the total number of predictions.
  27 $$\begin{aligned} Accuracy = \frac{TP+TN}{TP+TN+FP+FN} \end{aligned}$$
- Precision is calculated as the ratio of the number of true positive samples to the total number of samples classified as positive (correctly or incorrectly). Precision is used to evaluate the model’s effectiveness in classifying positive samples.
  28 $$\begin{aligned} Precision = \frac{TP}{TP+FP} \end{aligned}$$
- Recall is calculated as the ratio of the number of true positive samples to the total number of positive samples. The Recall only considers how positive samples are classified and is entirely independent of the classification of negative samples.
  29 $$\begin{aligned} Recall = \frac{TP}{TP+FN} \end{aligned}$$

If the model’s classification result has many False Positives or few True Positives, the Precision value will be small. If the model classifies all positive samples as positive, then Recall will always have a value of 100%, even if all negative samples are incorrectly classified as positive. Therefore, depending on the specific requirements of each case, the values of Accuracy, Precision, and Recall will be chosen to evaluate the effectiveness of the classification system. In this research, with the aim of quickly assessing classification results for the initial screening of marine bioacoustic signals, the accuracy parameter is utilized for a rapid evaluation. Precision and recall parameters will be employed in subsequent studies to further enhance system quality.

### Classification results using STFT and STFT-CSI on actual dataset

**Figure 14:**
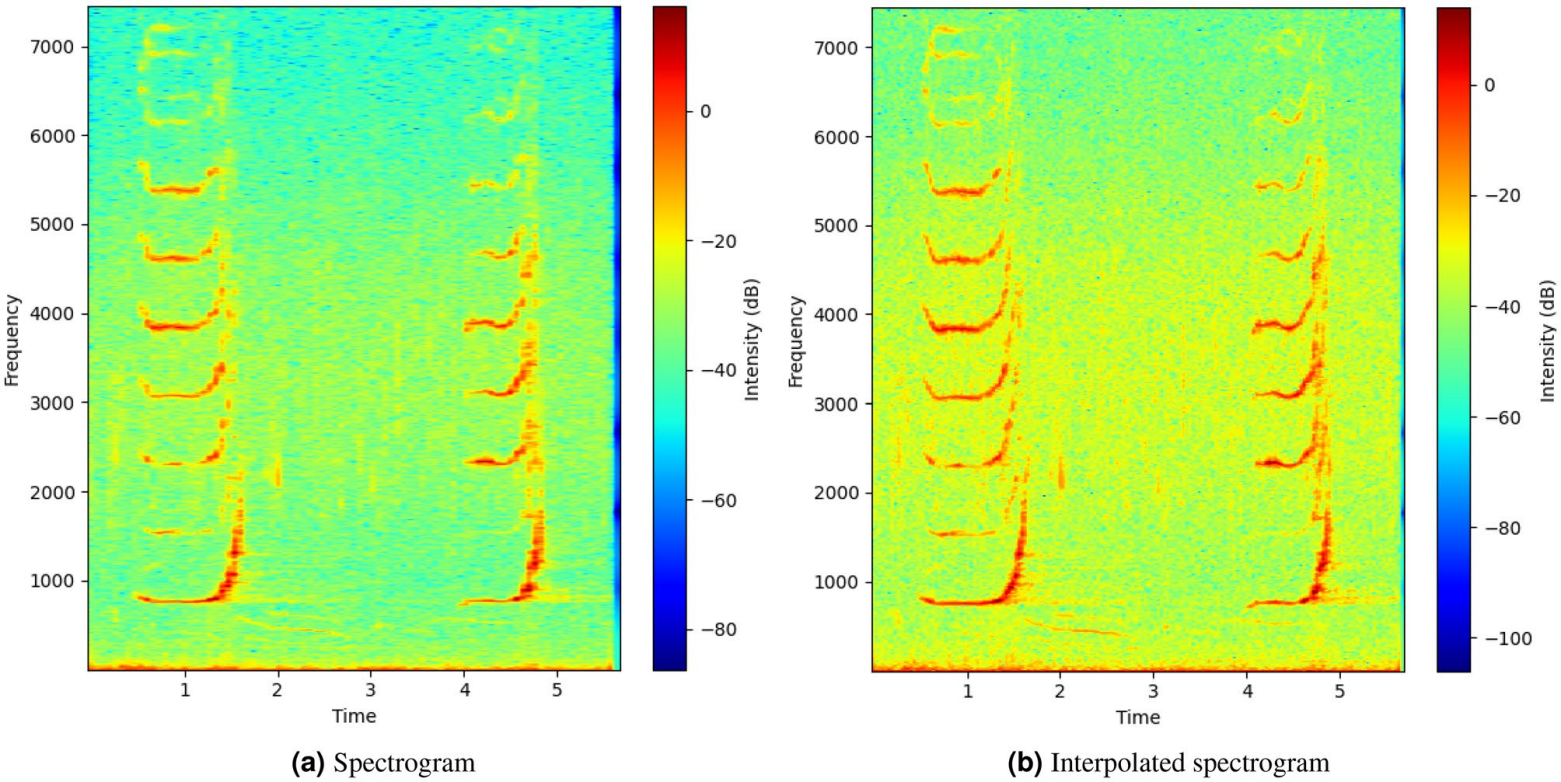
Spectrograms of Humback Whale signal from BOC-NOAA dataset.

To evaluate the effectiveness of feature extraction for marine mammals’ communication signals in practice (from the BOC-NOAA dataset^68^), the proposed Cubic-splines interpolation algorithm in Fig. 7 and the CNN structure (which was published in 2022^38,58^) will be utilized with equivalent parameters to train data with and without interpolation. The paper uses four groups of species from BOC-NOAA dataset: Common Dolphin (CD), Spinner Dolphin (SD), Humpback Whale (HW), and Killer Whale (KW), which are four typical marine mammals not only found in shallow waters of the United States but also of Vietnam^70^. The pre-processing of data, training, and testing were performed using an Ubuntu 18.04 operating system with a CUDA 10.1 and Cudnn 7.6.5 on a Dell T3600 Xeon 8-core workstation with an NVIDIA k2200 4GB graphics card. The input are fully RGB spectrograms with the size of (224x224), generated from the STFT with a Hanning window, an FFT size of 256, and 75% overlapping. Figure 14a and b represent samples of humpback whale signals with and without CSI method.

The paper divides data into 5-second records, ensuring that each record contains more than one fundamental frequency to verify the proposed interpolation algorithm. The interpolated and non-interpolated spectrogram are split separately into two sets of images which are inputs to the customized CNN^38^ for classification. When the data is limited in quantity and there are variations in length between the classes, the cross-validation process is used to ensure randomness in feature estimation and to limit statistical errors in classification. The spectrograms are randomly divided into *K* equal-sized subsets. Only one subset is kept as validation data, and $K-1$ subsets are used for training. Then, the cross-validation process is repeated *K* times, with each subset being used only once. The results obtained from each process are combined to provide the final classification accuracy result. The model in this paper uses cross-validation with *K*=5. The dataset is divided into training, validation, and test sets with respective proportions of 70%, 20%, and 10%.

**Figure 15:**
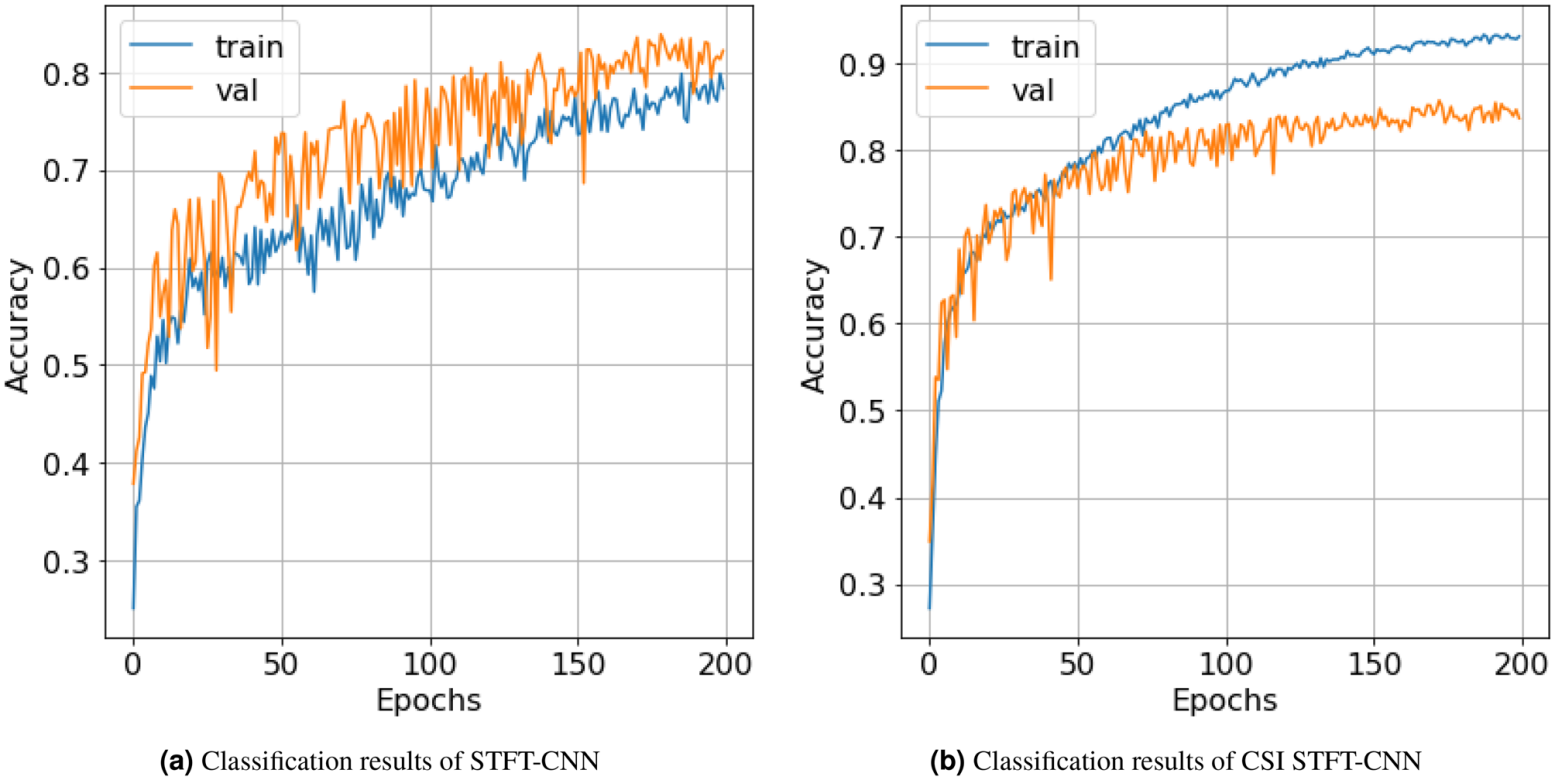
Comparison of the classification results between using and not using Cubic-splines interpolation.

**Figure 16:**
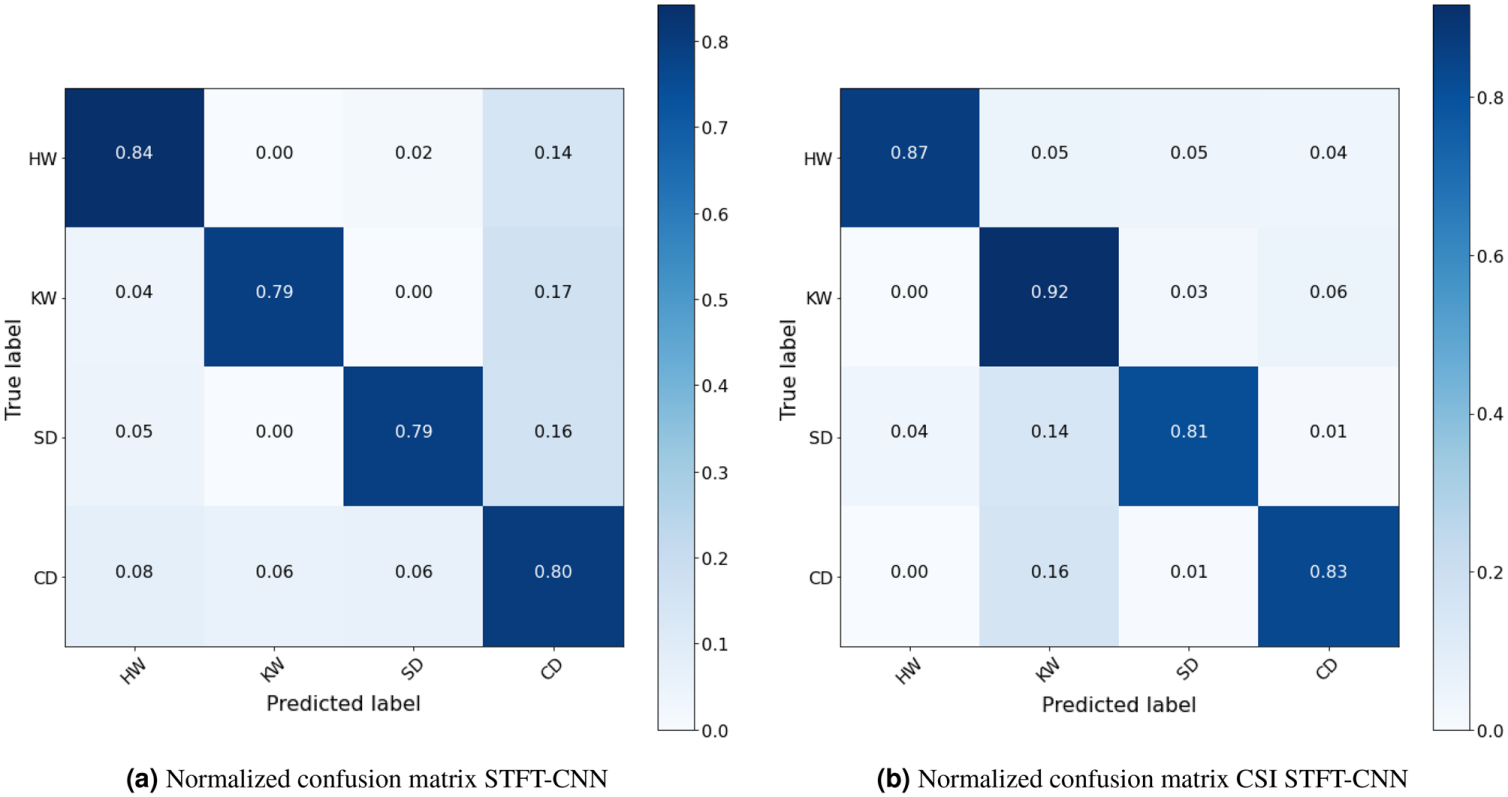
Comparison of normalized confusion matrixes between using and not using CSI with STFT-CNN model.

Comparing the accuracy of the pre-processing solutions using STFT and STFT-CSI method on the same CNN configuration, the proposed interpolation solution extracted fundamental frequencies better, resulting in increased classification accuracy and reduced underfitting. In addition to increasing the association between the characteristic frequencies, representing the “portrait” of the signal spectrum using interpolation also ensures that changes in characteristic frequencies do not occur suddenly. Thus, the CNN can learn all the properties of the spectrogram, thereby improve classification accuracy. The results in Fig. 15a show that the proposed CNN model achieves an accuracy rate of 80.5%, but does not generalize the relationship within the entire dataset, leading to a higher validation score than training score. Meanwhile, the cubic-splines interpolation (CSI) solution increases the accuracy rate by an additional 6%, reaching 86.5%, as shown in Fig. 15b.

The proposed solution using STFT interpolation for pre-processing has a higher accuracy than the published method without the STFT interpolation on the same NOAA dataset, as shown in Table 2.

**Table 2:** Comparison of the proposed CSI with published results on the same actual NOAA dataset.

	Proposed of paper	Related result, 2022	Related result, 2022^58^
Pre-processing	**CSI-STFT**	STFT	STFT
Network model	CNN	CNN^71^	CNN^58^
Classification Accuracy	**86.5%**	80.5%	80.45%

Significant values are in bold.

The normalized confusion matrixes in Fig. 16 represents the classification accuracy of each class of marine mammal signals. The classification results of each class in Fig. 16b and a have demonstrated the effectiveness of the cubic-splines interpolation algorithm on actual signals, particularly for the Killer Whale (KW) signals when the recorded data was limited to only 94 seconds (as shown in Table 1). The proposed interpolation algorithm shows a significant increase in classification accuracy from 79% to 92%. However, the classification results also indicate that using only STFT transformation does not fully capture the naturally complex and random features of marine mammals communication dataset. Therefore, the paper additionally proposes the use of three transformations STFT, Mel and Wavelet to further improve the quality of marine mammal signal classification.

### Classification results using the combination of two proposed solutions on marine mammal signals

To evaluate the effectiveness of the pre-processing solution using CSI combined with the SNN-VAE using probabilistic distribution in the latent space, the actual dataset NOAA^66^ will be used as input to evaluate the proposed solution with the same size and configuration parameters. The using dataset contain signals of four classes: Common Dolphin (CD), Spinner Dolphin (SD), Humpback Whale (HW), and Killer Whale (KW), are split into 5-second-segment data.

The datasets used for training, validation, and testing are divided into a ratio of 70-20-10. Each training batch consists of 256 samples with a total of 200 epochs. All processes are conducted in the Ubuntu 18.04 operating system with CUDA 10.1 and Cudnn 7.6.5 on a Dell T3600 Xeon 8-core workstation equipped with an NVIDIA k2200 4GB graphics card.

#### The first case: evaluate the effectiveness of each method

**Figure 17:**
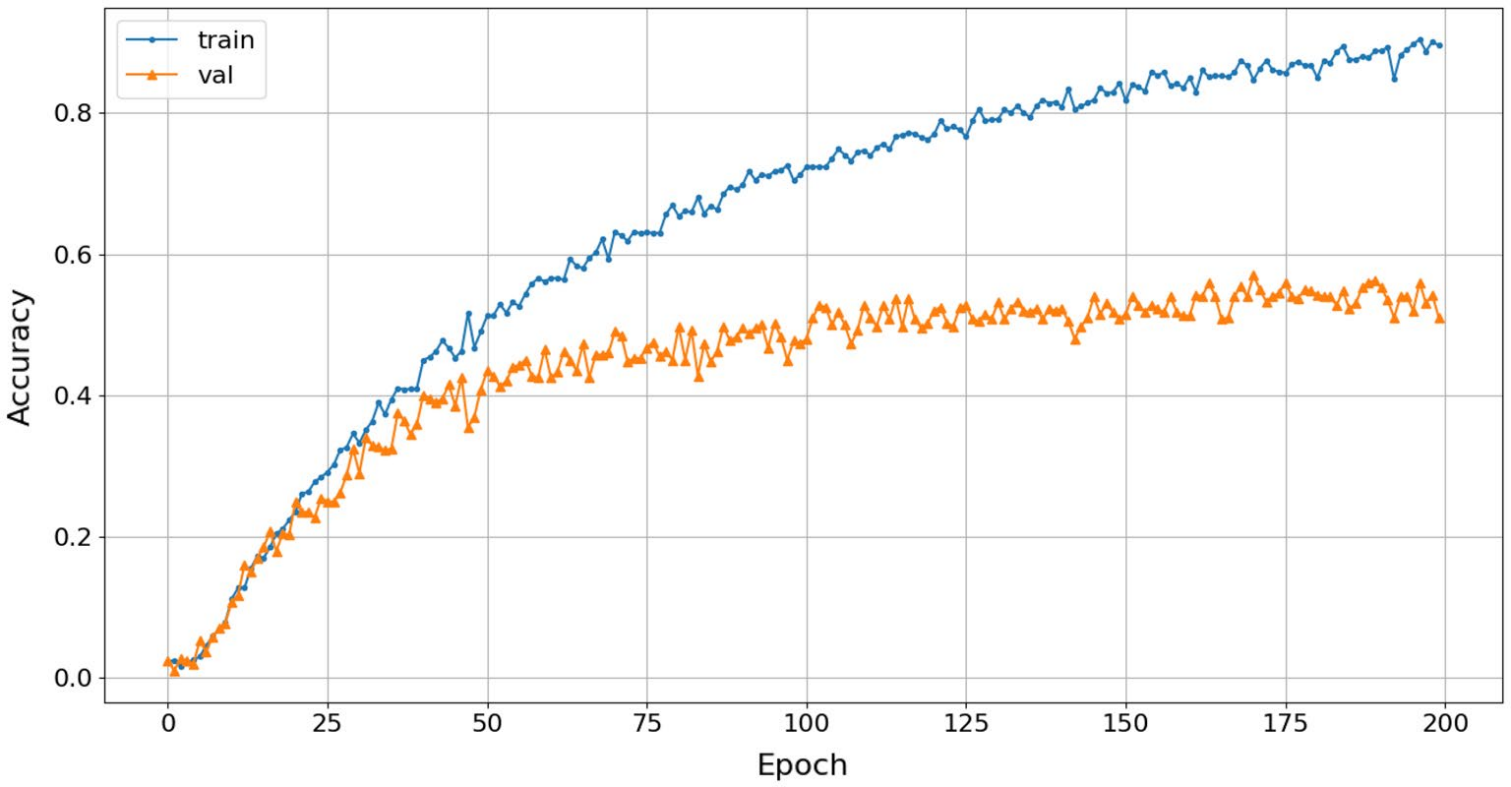
Training accuracy of STFT-Rep-VGG-A0.

From the NOAA dataset, spectrograms are created using the STFT with a Hanning window function, a 256-sample FFT window, and a 75% overlap. The Scalogram is created using the Wavelet transform with the Haar symmetric function and a 512-sample window length. The Mel-spectrogram is created using the MFCC with a 1024-sample window length and 64 mel frequency bands. The accuracy of the 4-class marine mammals classification using STFT pre-processing and the Reg-VGG-A0 model achieved a result of 52%, as shown in Fig. 17. The accuracy of the 4-class marine mammals classification using STFT pre-processing and the Reg-VGG-A0 model achieved a result of 52%, as shown in Fig. 17.

**Figure 18:**
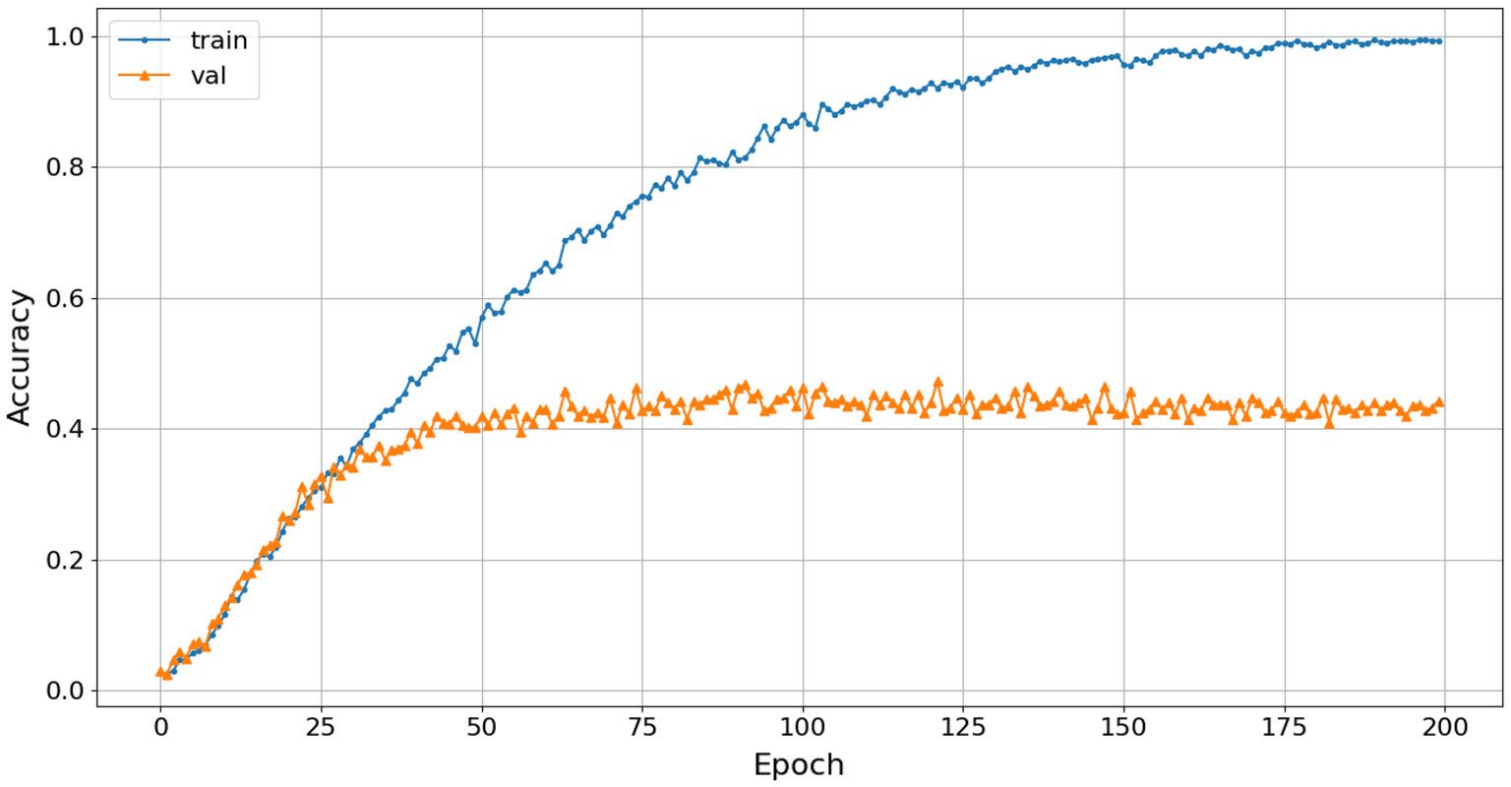
Training accuracy of Wavelet-Rep-VGG-A0.

The accuracy of the 4-class marine mammals classification using Wavelet pre-processing and the Reg-VGG-A0 model achieved a result of 45%, as shown in Fig. 18.

**Figure 19:**
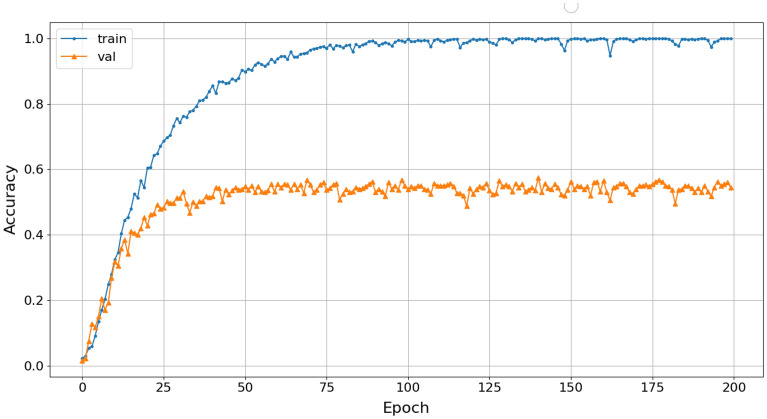
Training accuracy of Mel-Rep-VGG-A0.

The accuracy of the 4-class marine mammals classification using Mel pre-processing and the Reg-VGG-A0 model achieved a result of 58%, as shown in Fig. 19.

The classification results show a significant change in the accuracy of the spectrogram, scalogram, and Mel-spectrogram datasets during training with the Rep-VGG-A0, as well as early saturation of the model. The Rep-VGG model has a deep convolution structure, but the network does not extract signal features effectively. Those results show that the pre-processing with STFT, Wavelet, and Mel transforms faces certain limitations in analyzing the data structure of the long term actual datasets collected as NOAA dataset.

#### The second case: using cubic-splines interpolation

In order to overcome the limitations of using individual methods with regards to resolution, a solution utilizing simultaneous STFT, Wavelet, and Mel can provide greater flexibility in changing the time and frequency resolution of the approach. The use of multiple types of spectrogram with different transorms are particularly effective in processing biotic data containing signals from different marine mammals, as the low-frequency signals emitted by whales tend to be long-lasting and narrow-bandwidth^72^, while the high-frequency signals emitted by dolphins tend to have a wider bandwidth and higher frequency^73^. Therefore, for actual data with prolonged recording times as the NOAA, individual techniques are highly susceptible to mis-classifying signals from different classes that has the same frequency.

**Figure 20:**
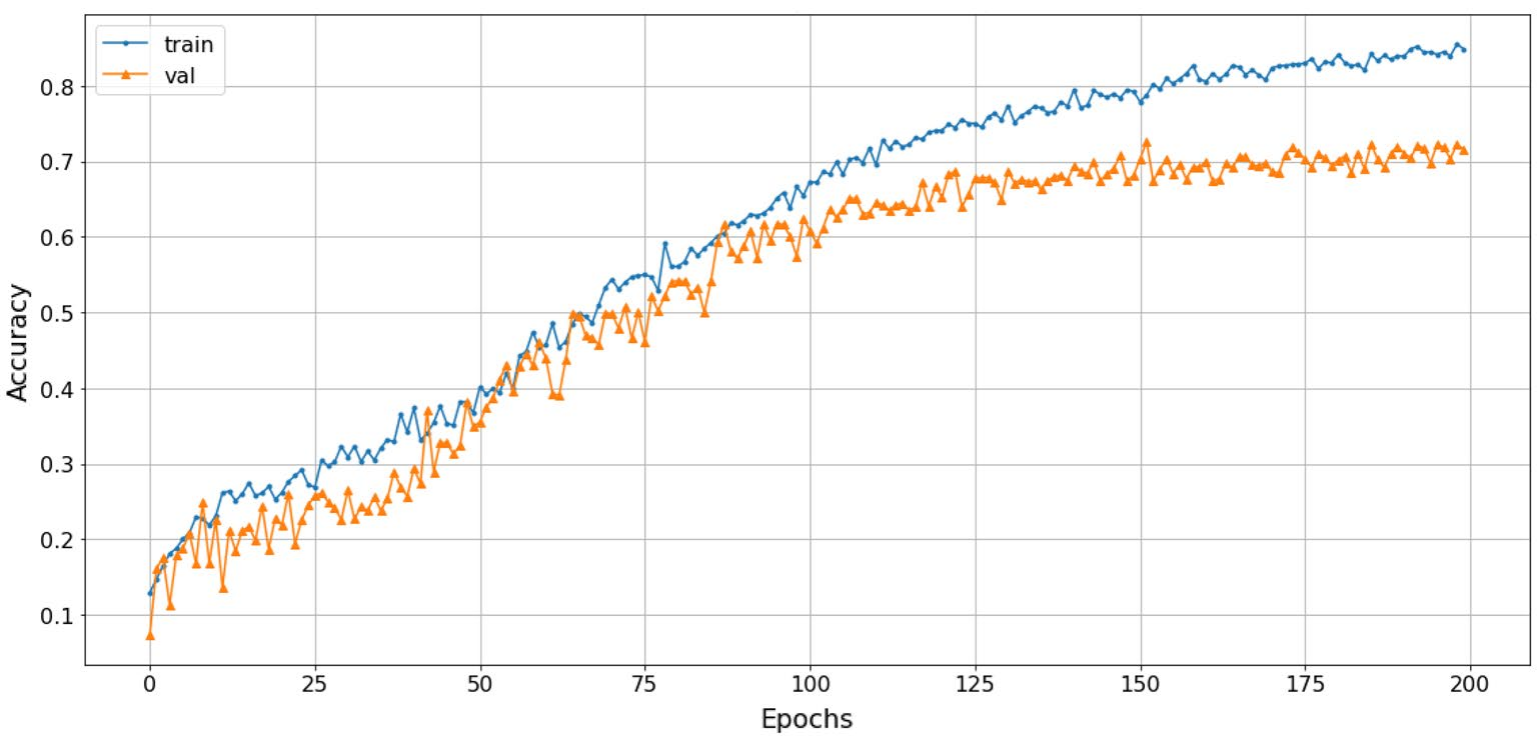
The accuracy of stacked CSI with Rep-VGG-A0 model.

**Figure 21:**
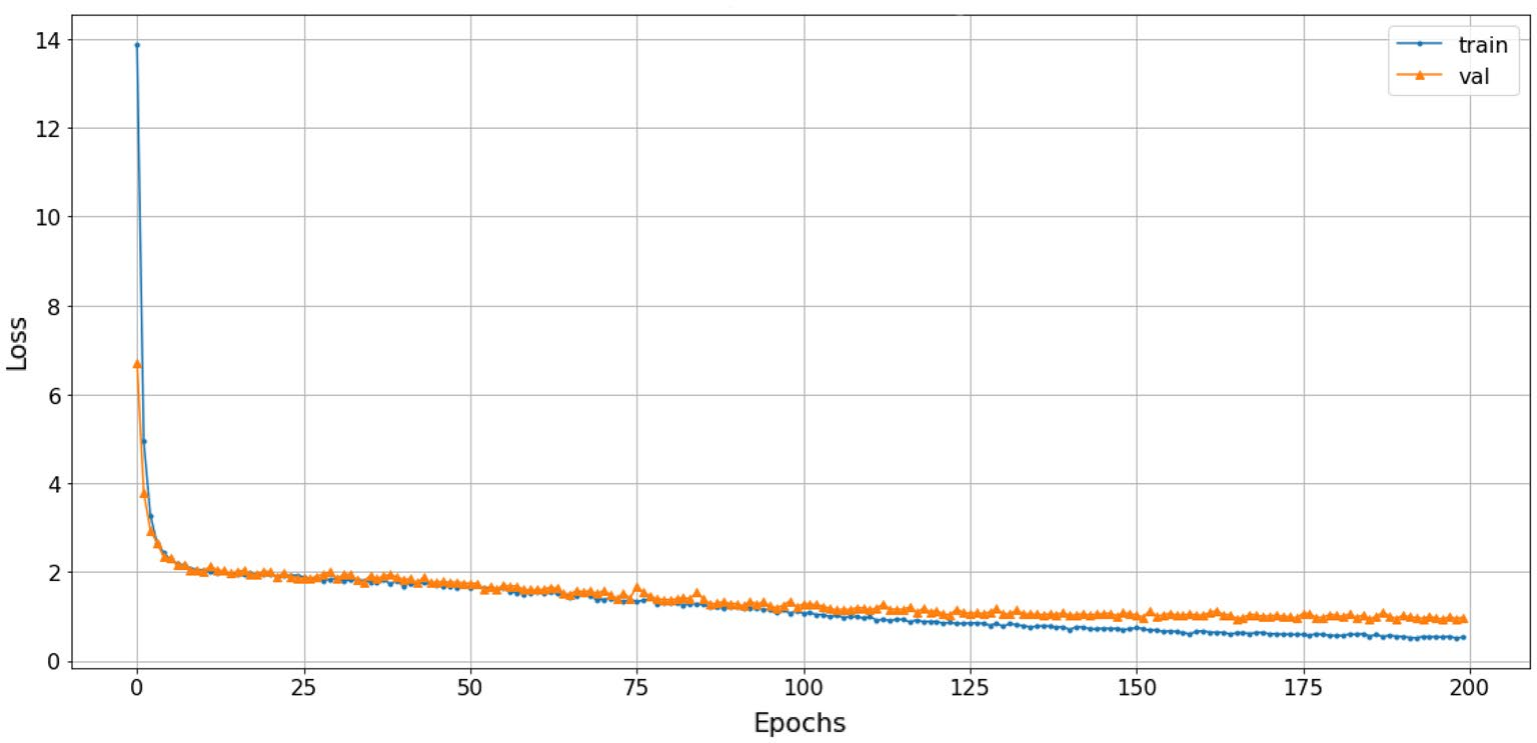
The loss of stacked CSI with Rep-VGG-A0 model.

The proposed solution involves stacking spectrograms generated from three transformations with cubic-splines interpolation, which achieves a classification accuracy of approximately 72% in Fig. 20, higher than when using individual techniques, and avoids saturation under the same training conditions. The loss function value converges to 1 in Fig. 21.

**Table 3:** Distribution marine mammal classes in fully NOAA dataset.

	Common dolphin	Spinner dolphin	Humpback whale	Killer whale
Amount	604 (records)	692 (records)	524 (records)	684 (records)
Duration	935 (s)	838 (s)	1025 (s)	662 (s)

For the fully NOAA dataset in Table 3, the number of recordings for Humpback whales, Killer whales, Spinner dolphins, and Common dolphins are 604, 692, 524, and 684, respectively. These recordings have varying qualities and are collected at different times, with significant background noise. Once again, this confirms that using a single time-frequency domain transformation method presents several limitations when dealing with long-term recordings and background noise.

#### The third case: replacing Rep-VGG-A0 by SNN-VAE

The study employed a stacked combination of spectrogram, scalogram, and mel-spectrogram with cubic-splines interpolation pre-processing. This approach exploited the advantages of each technique and improved the processing quality by extracting all relevant features, highlighting the relationships between extracted frequencies, and enabling the proposed model to learn more useful information. The loss function gradually converged to an approximate value of 1. The efficiency of the SNN-VAE and Rep-VGG-A0 were evaluated by the convergence of loss function, accuracy and confusion matrix.

**Figure 22:**
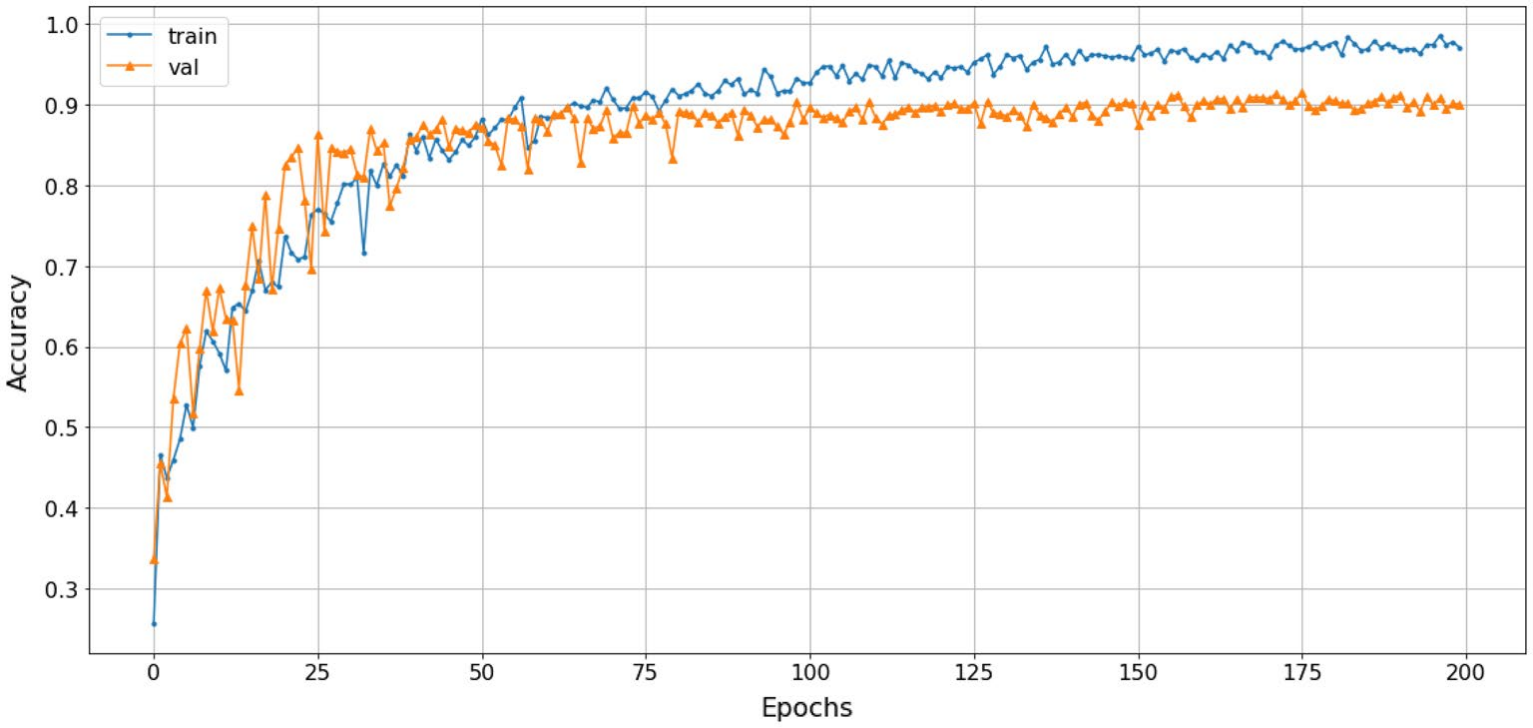
The accuracy of stacked CSI with SNN-VAE model.

**Figure 23:**
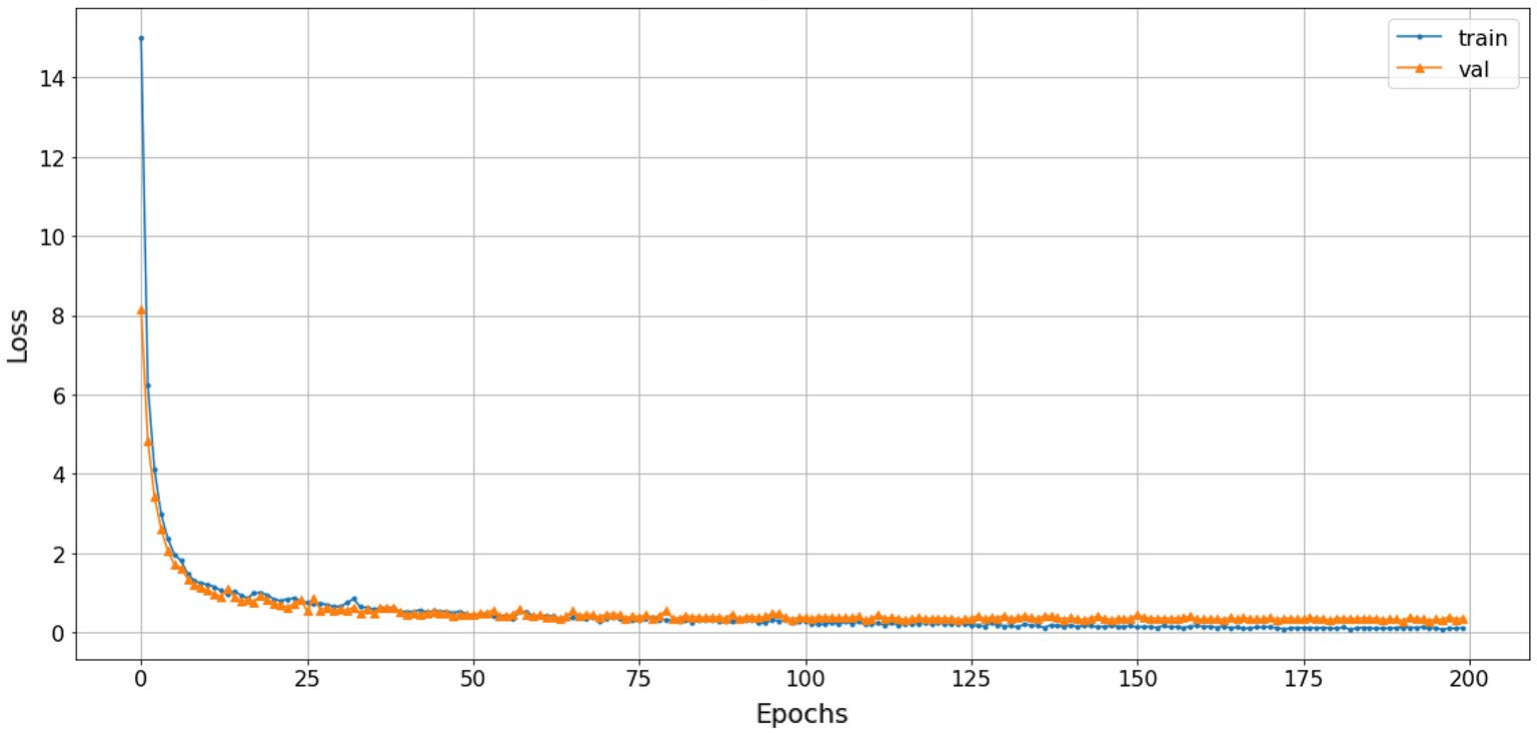
The loss of stacked CSI with SNN-VAE model.

The loss function of the SNN-VAE decreased significantly compared to the Rep-VGG (Figs. 21 and 23) when both models were trained for 200 epochs. The combination of stable data spacing, data restructuring as a distribution, and the deep multi-layer convolution characteristics of the SNN-VAE helped to improve accuracy, prevent overfitting and underfitting; and reduce the convergence of the loss function below 0.5, compared to 1 when using the Rep-VGG-A0. The classification accuracy of marine mammal signals increased by nearly 20%, from 72 to 91.2%, when classifying four classes of marine mammals as shown in Fig. 22. The loss function value converges to 0.5 in Fig. 23.

**Figure 24:**
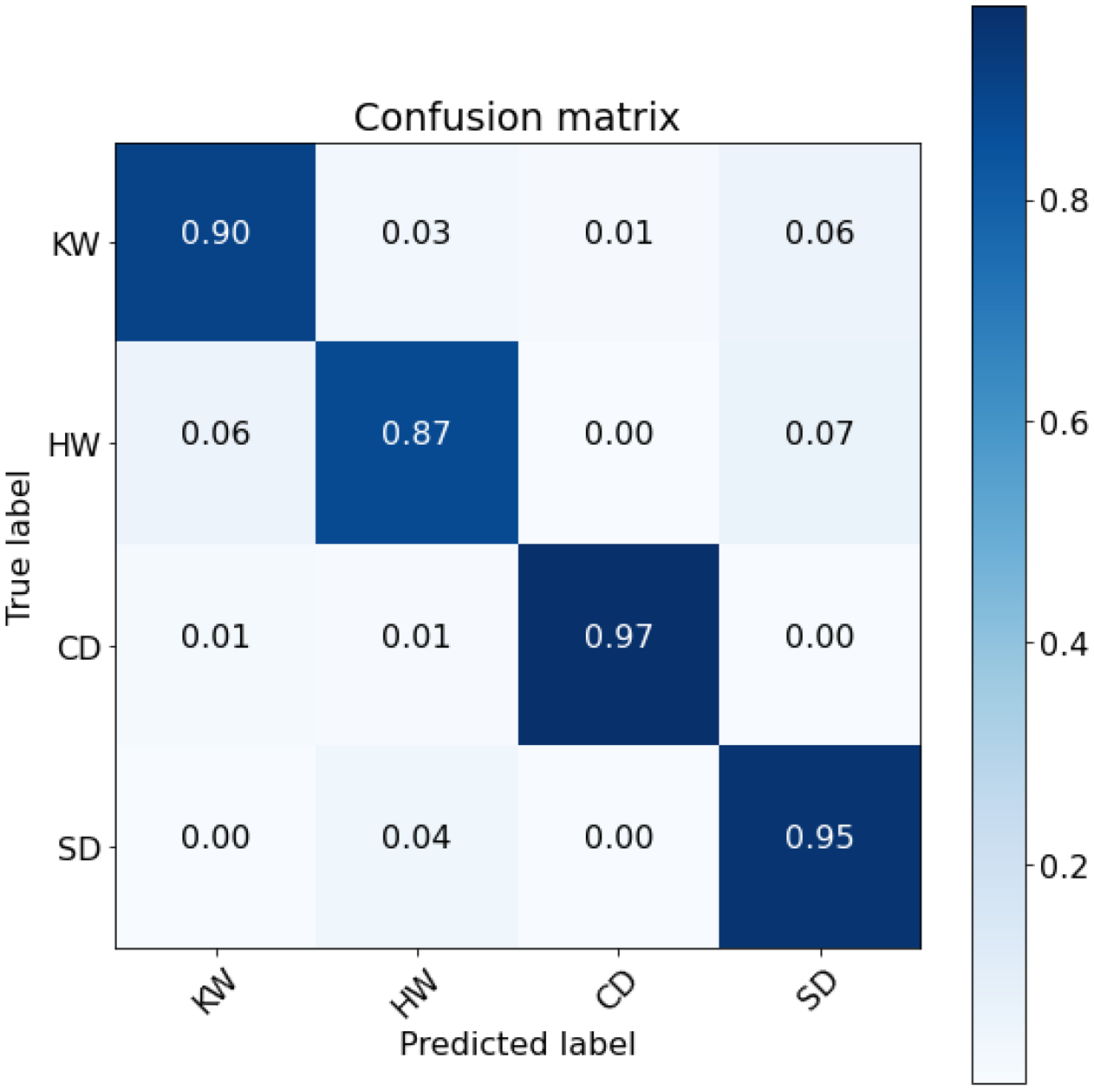
Confusion matrix of 4-class marine mammals classification.

The classification results for each categories of 4-class marine mammals using the proposed SNN-VAE were represented in a confusion matrix (Fig. 24). The stacked spectrogram with SNN-VAE achieved classification accuracy rates of 90%, 87%, 97%, and 95% for KW, HW, CD, and SD, respectively. These results were higher than those obtained by NOAA’s BOC classification solutions, which only used STFT combined with customized CNN networks (84%, 79%, 79%, and 80%) or STFT interpolation combined with custom CNN networks (87%, 92%, 81%, and 83%).

**Table 4:** Comparing with published results using the same NOAA dataset.

	proposed SNN-VAE	Rep-VGG-A0	AE^74^	ResNet^58^
Accuracy	**91.2%**	72%	Noise: 69% No noise: 80%	85.4%

Significant values are in bold.

The average accuracy result of 91.2% (as shown in Table 4) demonstrates that the proposed model has improved compared to other Rep-VGG in general (72%), the Auto-Encoder (AE) model^74^ (69% and 80% respectively with noise and non-noise data), and the ResNet model^58^ (85.4%). The proposed solution achieved an accuracy rate 11% higher than the AE structure model published on the same dataset. This further confirms the effectiveness of the VAE distribution encoding solution over the conventional Auto-Encoder when extracting information from actual data with complex background noise and overlapping frequency bands.

### Classification results of the proposed cubic-splines interpolation and SNN-VAE on marine mammal and propeller signals

The datasets from NOAA^66^ and DeepShip^33^ were used to evaluate the effectiveness of the proposed pre-processing solution using cubic-splines interpolation combined with the probability distribution in latent space of SNN-VAE for input consisting of multiple biotic and abiotic signals. The input data for the SNN-VAE model comprised: interpolated spectrograms, scalograms, and mel-spectrograms of the marine mammals as used in The Third case section and Lofargrams of the propeller ships^71^

The dataset consisted of four classes of marine mammals and two classes of propeller ship, specifically:

- Signals from four species of marine mammals: Common Dolphin (CD), Spinner Dolphin (SD), Humpback Whale (HW), and Killer Whale (KW), were segmented into 5-second records.
- Signals from two classes of propeller ships: Cargo (C) and Passenger (P) were segmented into 200-second records.

The datasets used for training, validation, and testing were divided in a 70-20-10 ratio. The batch size for each training iteration was set to 256, and the number of training iterations was set to 200. The pre-processing were performed on a Dell T3600 Xeon 8-core workstation with an NVIDIA k2200 4GB graphics card running Ubuntu 18.04 with CUDA 10.1 and Cudnn 7.6.5.

**Figure 25:**
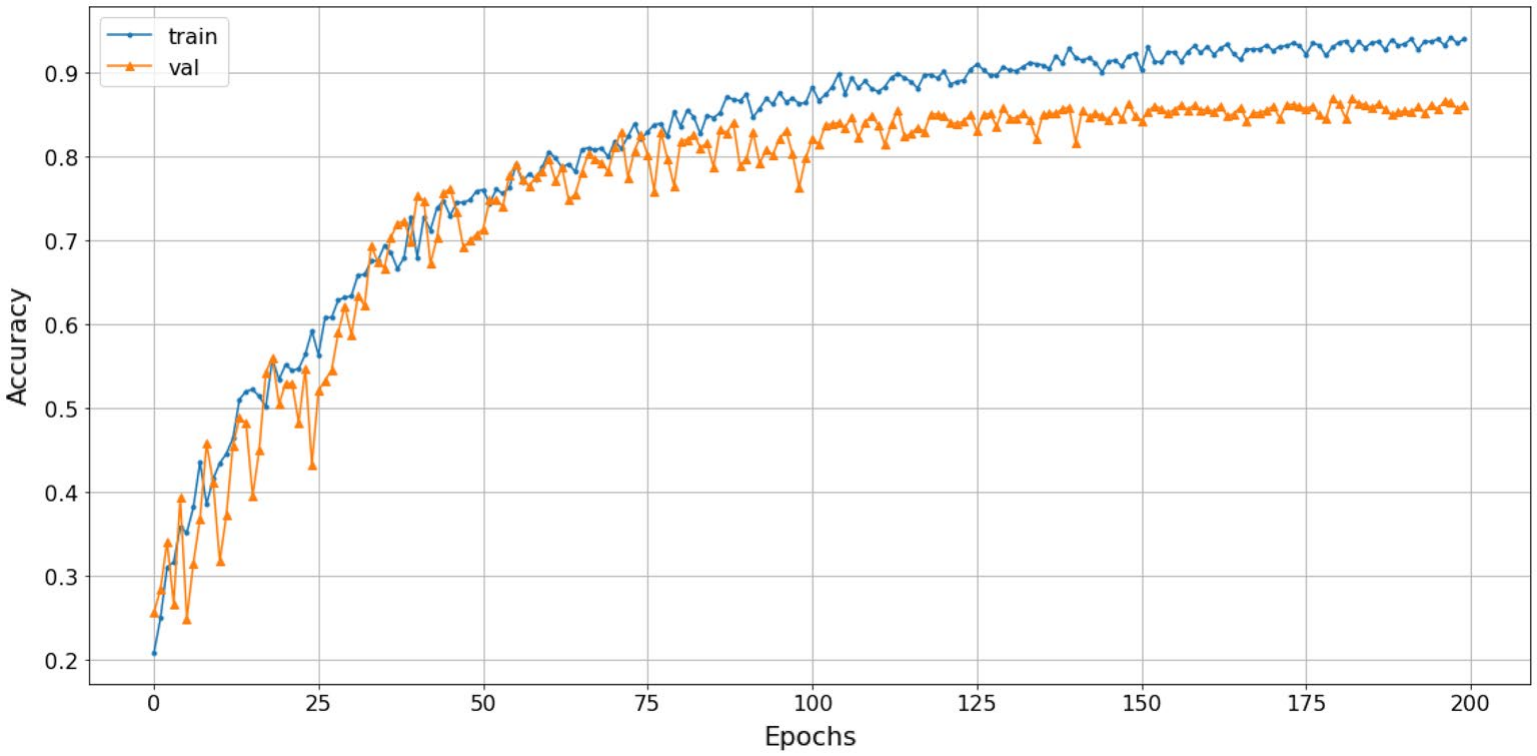
The accuracy of SNN-VAE with 6 classes.

**Figure 26:**
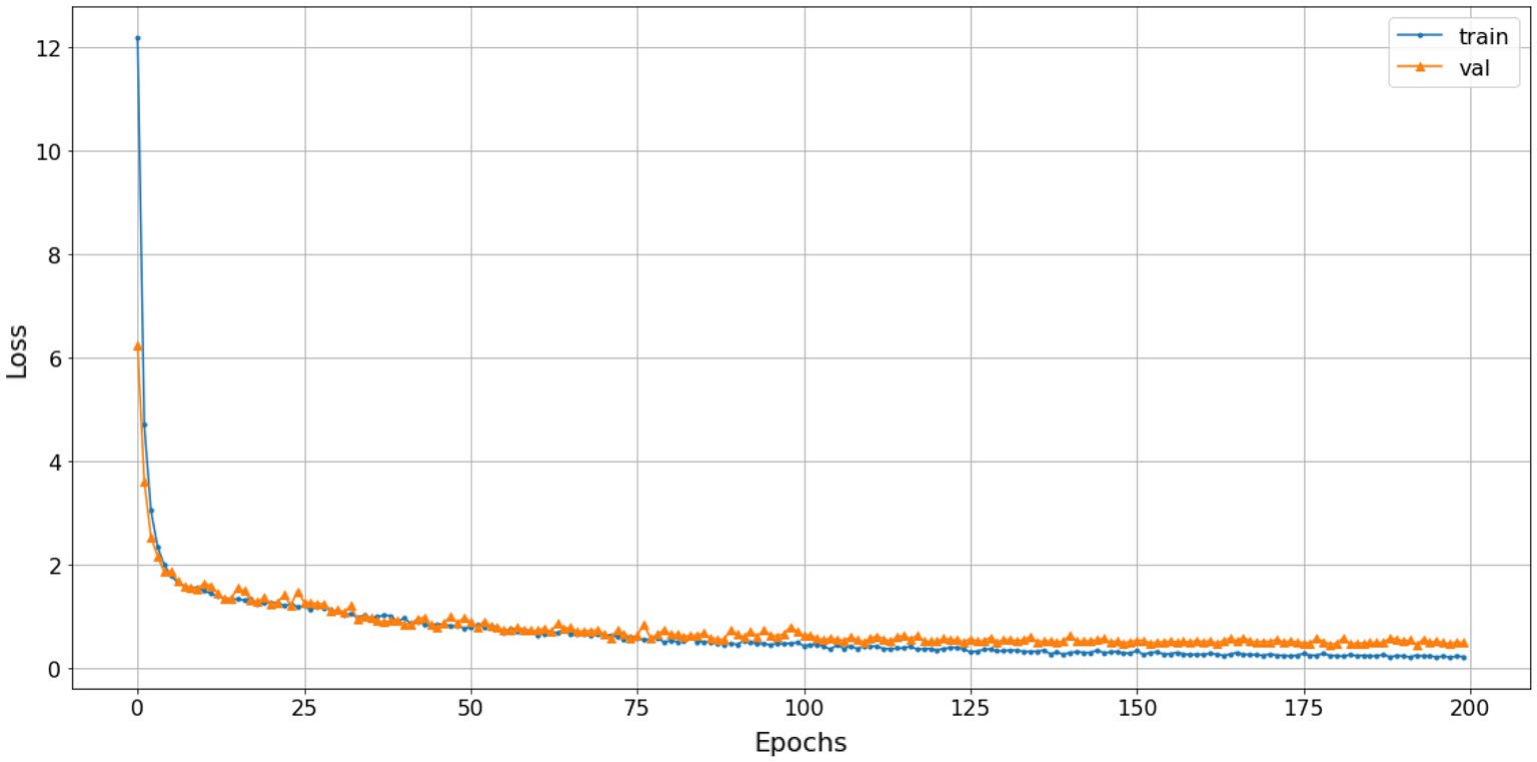
The loss of SNN-VAE with 6 classes.

**Figure 27:**
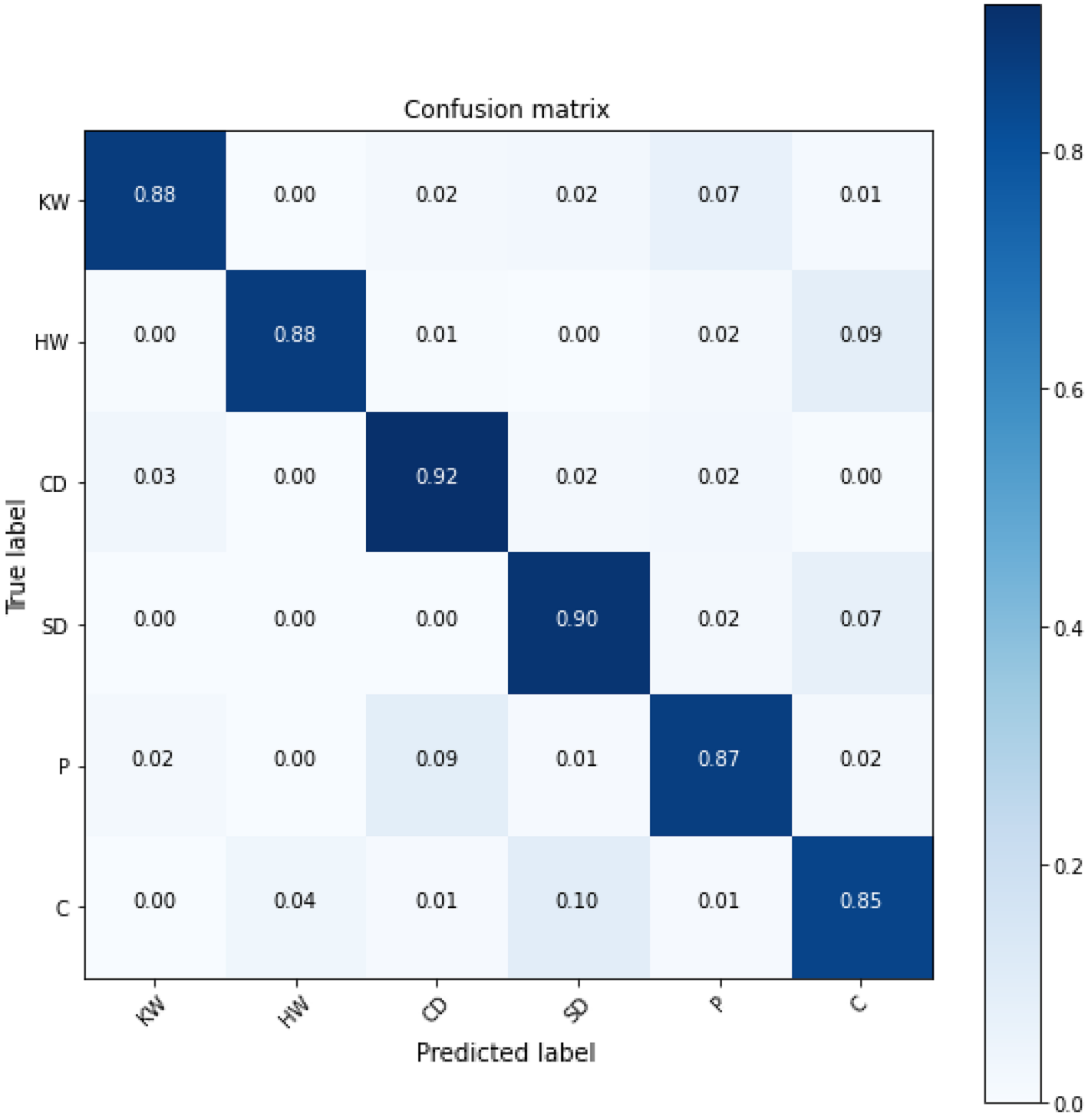
Confusion matrix of CSI combined with SNN-VAE for 6 classes.

The classification accuracy and loss function of the SNN-VAE model are presented in Figs.  25 and 26 (loss function value converges to 0.55), respectively, while the confusion matrix illustrating the classification results of the six signal classes is presented in Fig. 27.

The proposed SNN-VAE model achieved high accuracy in classifying different groups of marine mammals and propeller ships, with classification accuracy of 88%, 88%, 92%, 90%, 87%, and 85% respectively (Fig. 27). The average accuracy of the SNN-VAE network was 89.5% (Fig.  25) in classifying six groups of objects, while the probability of correctly detecting signals from biotic signals decreased by about 1% compared to when classifying only four groups of objects with marine mammal signals (91.2%, Fig. 22). The classification results of Cargo and Passenger ships in the combined object group were 85% and 87%, respectively, which were equivalent to the results of classifying only propeller ships which were published in^71^. Therefore, the application of the SNN-VAE structure to classify underwater data records containing both biotic and abiotic signals is reasonable. The improvement in accuracy is attributed to the normalization of the covariance matrix and mean of the distribution, which limits the separation of encoded distributions and encourages overlapping distributions to make the model more continuous and complete.

In general, the proposed SNN-VAE model employs a three sub-network convolution structure combined with probability distribution in the latent space compared to traditional VGG (2016) or modern Rep-VGG (2022) convolution networks that have achieved certain efficiencies, improving the accuracy of classifying real signals in the agricultural water environment. The following are the main points of focus:

*The first* by encoding the input data into a lower-dimensional representation to identify important features, the SNN-VAE model has the ability to improve classification accuracy compared to using raw input data directly. In addition, SNN-VAE can still handle incomplete or missing underwater data, as the probability space of SNN-VAE always generates meaningful representations even when input data points are missing. This feature is effective in real-world conditions where the quality of the recorded signals is poor.

*The second* the SNN-VAE network can be trained using unsupervised learning techniques, which has many advantages when labeled training data is limited. Furthermore, unlike the approach of traditional convolutional networks that always attempt to extract the most common features of the data, the SNN-VAE network structure uses identical sub-networks to compare the differences between inputs. The SNN-VAE network will rely on the differences between each pair of data to produce classification results, making it still perform well even with limited hydroacoustic datasets.

*The third* the loss function ${\mathscr {L}}_{SNN-VAE}$ of SNN-VAE is the sum of stable loss functions ${\mathscr {L}}(\textbf{A, P, N})$, reconstruction loss ${\mathscr {L}}_{reconstruct}$, and Kullback-Leibler divergence $\beta {\textbf{K}}{\textbf{L}}(z, N(0, I_d))$, making the model more flexible during training. Depending on the requirements of the classification problem, the model will focus on using and optimizing different loss functions.

The classification results of the proposed model for each data group containing marine mammal signals and mixed two signal groups (marine mammal and propeller signals) are presented in Table 5 as follows:

**Table 5:** Classification results of each deployed model with biotic and abiotic signals.

Pre-processing	Network	Target	Data	Accuracy	Figure
STFT	CNN^71^	Marine mammals	BOC-NOAA	80.5%	15a
Interpolated STFT-CSI(*)	CNN^71^	Marine mammals	BOC-NOAA	86.5%	15b
STFT	Rep-VGG^65^	Marine mammals	NOAA	52%	17
Mel	Rep-VGG^65^	Marine mammals	NOAA	45%	19
Wavelet	Rep-VGG^65^	Marine mammals	NOAA	58%	18
Interpolated stack(*) (STFT-CSI,Mel and Wavelet)	Rep-VGG^65^	Marine mammals	NOAA	72% Loss: 1	21
Interpolated stack(*) (STFT-CSI,Mel and Wavelet)	SNN-VAE(*)	Marine mammals	NOAA	91.2% Loss: 0.5	23, 22
Interpolated stack(*) (STFT-CSI,Mel and Wavelet)	SNN-VAE(*)	Marine mammals, Propeller ships	NOAA, Deepship	89.5% Loss: 0.55	25, 26

$^{(*)}$ Proposed solution of the paper.

## Conclusions

This paper presents a method for detecting and classifying real underwater acoustic signals in a marine environment, including duck sound signals and communication signals from marine mammals, in the presence of background noise. The research results are based on the cubic-splines interpolation to enhance the spectrogram quality after pre-processing step, combined with the proposed SNN-VAE model, achieving an accuracy of approximately 90% on actual datasets containing complex signal components. By using high-degree polynomial mathematical transformations, the cubic-splines interpolation are applied to underwater acoustic signals analysis to increase their connectivity, discover relationships between characteristic frequency components without changing the signal structure. In addition, the deep learning network model uses a probability distribution on the hidden space domain to ensure the continuous and stable classification model, enhance the ability to extract accurate and sufficient features from the underwater signal. The next directions for development involve applying interpolation algorithms to other time-frequency transformations such as Mel, Wavelet, DEMON (Demodulation of Envelope Modulation On Noise), and LOFAR (Low-Frequency Analysis and Recording) to evaluate the specific algorithm’s effectiveness in practical situations.

## Data Availability

The datasets generated and analysed during the current study are available in the website of National Oceanic and Atmospheric Administration, U.S. Department of Commerce [https://www.noaa.gov/] and The Woods Hole Oceanographic Institution [https://cis.whoi.edu/science/B/whalesounds/index.cfm].
